# Distinct immune microenvironments in ovarian cancer subtypes indicate potential for immunotherapies

**DOI:** 10.1186/s12967-026-07879-8

**Published:** 2026-02-21

**Authors:** Asger Meldgaard Frank, Elias Carlsson, Huaqiang Ouyang, Lina Olsson, Constantina Claudia Mateoiu, Sara Ek, Karin Sundfeldt, Anna Gerdtsson

**Affiliations:** 1 Department of Immunotechnology, Lund University, Medicon Village 406, Scheelevägen 8, Lund, 223 87 Sweden; 2 Department of Integrative Oncology, Tianjin Medical University Cancer Institute and Hospital, West Huan-Hu Rd, Ti Yuan Bei, Hexi District, Tianjin, 300060 P. R. China; 3 Department of Pathology and Cytology, Sahlgrenska Academy at University of Gothenburg, Gula stråket 8 plan 0, Göteborg, 41345 Sweden; 4 Department of Obstetrics and Gynecology, Sahlgrenska Academy at University of Gothenburg, Sahlgrenska Hospital, Blå stråket 6 plan 2, Kvinnokliniken, Göteborg, 41345 Sweden

**Keywords:** Ovarian cancer, Tumor microenvironment, Immune-oncology, Spatial biology, Digital spatial profiling

## Abstract

**Background:**

To enable immunotherapy for ovarian cancers, precision targets for immune priming as well as patient stratification approaches are required. The tumor microenvironment in particularly the rare subtypes of low-grade serous, mucinous, clear cell and endometrioid ovarian cancer, remains poorly characterized, and these tumors have been largely ignored in the immuno-oncology setting.

**Methods:**

We performed spatially resolved molecular profiling of 78 tumor and immune protein markers in defined tissue regions from 254 ovarian cancer patients of mixed histologies, using GeoMx. Network graph analysis was applied to compute spatial statistics from multiplex immunofluorescence images. GeoMx-compatible softwares were developed for data processing and analysis, based on linear mixed effect modelling, survival analysis and machine learning.

**Results:**

Immune-regulatory targets associated with specific subtypes included STING in low-grade serous; CTLA-4 and PD-L1 in mucinous; CD40, IDO1 and VISTA in clear cell; and B7-H3 in endometrioid ovarian carcinomas. In high-grade serous ovarian carcinomas, intra-tumoral expression of SMA and PD-L1 emerged as strong prognostic indicators. Proximity of CD8 + T-cells to tumor cells as measured by group degree centrality was a marker of improved prognosis and of infiltration of all T cell subtypes, dendritic cells, and tumor-associated macrophages, along with elevated expression of PD-L1, IDO1, Tim-3, and CD40. In contrast, tumors with low CD8-tumor proximity were enriched in CD20 and CD25.

**Conclusions:**

Our findings highlight the potential for differential targeted treatment related to histotyping, tumor-immune spatial scoring and intra-tumoral expression of key prognostic markers.

**Supplementary Information:**

The online version contains supplementary material available at 10.1186/s12967-026-07879-8.

## Background

Ovarian carcinoma (OC) is a highly heterogeneous malignancy, yet most patients receive uniform treatment consisting of debulking surgery followed by platinum-based chemotherapy. The relapse rate is about 75% within two years from diagnosis for late stages, underscoring the urgent need for new precise and effective therapies to improve outcome [1]. Poly (ADP-ribose) polymerase (PARP) inhibitors have shown benefit for subsets of patients with homologous recombination deficient or *BRCA* variant tumors, however the long-term clinical efficacy remains limited [2].

OC is in general considered immunogenic, with the majority of tumors exhibiting infiltrating lymphocytes [3]. Nevertheless, the tumor microenvironment (TME) is complex and variable, often dominated by immunosuppressive and stromal components such as regulatory T-cells (Tregs), tumor-associated macrophages (TAMs) and cancer-associated fibroblasts (CAFs). This immunosuppressive milieu contributes to poor outcomes by limiting immunocytotoxicity and impeding therapeutic efficacy through mechanisms such as extracellular matrix remodeling and abnormal vasculature [4, 5].

CD8⁺ T cell infiltration has long been associated with improved survival in OC [6]. However, T cells may be rendered ineffective by various immune evasion strategies by cancer cells, including loss of MHC expression, impaired antigen presentation, recruitment of regulatory immune cells, and upregulation of immune checkpoints (e.g., PD-L1, B7-H3) and immunomodulatory enzymes (e.g., IDO1, ARG1) [7]. Unlike many other cancers, immune checkpoint inhibition (ICI) targeting PD-1/PD-L1 has yielded only modest responses in OC [8], despite reports of relatively frequent PD-L1 expression [9, 10]. Combination therapies involving ICI with anti-angiogenic agents or PARP inhibitors have shown improved response rates, yet overall efficacy remains low compared to many other tumor types [3, 11].

The limited success of immunotherapy in OC is largely attributed to its extensive interpatient heterogeneity and the absence of robust tools for stratifying patients based on TME profiles. Although *BRCA* mutations and homologous recombination deficiency status correlate with increased lymphocyte infiltration, neither is predictive of clinical benefit from ICI [12, 13]. Furthermore, most clinical trials have not accounted for histological subtype, despite vast differences in genetic background and presentation.

OC comprises five main histotypes: high-grade serous (HGSOC), low-grade serous (LGSOC), endometrioid (EOC), clear cell (CCOC), and mucinous (MOC) ovarian carcinoma. A dualistic classification into Type I and Type II OC is also used [14–16]. Type II tumors, which account for ~ 70% of cases and are predominantly HGSOC, are characterized by aggressive growth, late-stage presentation, and near universal *TP53* mutations. Type I tumors, including LGSOC, EOC, CCOC and MOC, each accounting for ~ 5–10% of OCs, tend to be slower-growing and harbor mutations in *KRAS*, *ARID1A*, *PIK3C*, *PTEN* and *BRAF*.

There are limited studies describing TMEs of the low-grade histotypes. While immunotherapy studies have primarily focused on HGSOC, emerging evidence suggests that subsets of patients with Type I histotypes may benefit from checkpoint inhibition [17]. In particular, treatment of CCOC has shown promise with combined PD-1 and CTLA-4 inhibition [17, 18], although durable responses remain limited. Recent data indicate that molecular subtypes within CCOC with improved response rate to ICI can be defined [19, 20]. For EOC, which shares endometriosis-associated features and expression profiles with CCOC but exhibits a less aggressive phenotype [21, 22], clinical data on immunotherapy efficacy are scarce. One study suggests that despite generally low immune infiltration, EOC may harbor a favorable TME due to a higher proportion of functional (non-exhausted) cytotoxic T-cells compared to HGSOC [23]. Conversely, MOC and LGSOC are generally poorly immunogenic, likely due to high frequency of *KRAS* mutations and *TP53* wildtype status associated with low tumor mutational burden [24, 25]. In MOC, dense stroma and abundant mucin production may further prevent immune infiltration and drug delivery.

Given the complexity of the OC TME and the multitude of cellular interaction influencing therapeutic outcomes, single biomarkers are unlikely to provide sufficient predictive power for immunotherapy response. To this end, spatial omics offer unprecedented capabilities to profile tumor and immune phenotypes with retained spatial context. We have previously employed GeoMx digital spatial profiling (DSP) combined with image-based spatial analysis to characterize spatially distinct TME regions in OC and shown that presence of immuno-oncology targets can be associated to infiltration patterns retrievable through spatial statistics [26].

In the present study, we expanded this approach to a substantially larger OC cohort to delineate key TME features across individual histotypes, with the aim to inform the potential for immune targeting in specific patient subpopulations and contribute to the development of more effective, stratified immunotherapeutic strategies.

## Materials and methods

### Patient cohort and TMA Preparation

The study was conducted in accordance with the Declaration of Helsinki. Tissue from 254 patients were analyzed on 12 tissue microarrays (TMAs), which were constructed from surgically resected tumors collected at the Sahlgrenska University Hospital between 2001 and 2015 (Regional Ethics Review Board in Gothenburg, reference 201 − 15)). Formalin-fixed paraffin-embedded (FFPE) biopsies were sectioned and stained with Hematoxylin and tumor areas were identified under light microscope. Three 1 mm diameter cores from each tumor were punched with a manual tissue microarrayer (Beecher MTA-1, Estrogen Tartu, Estonia) and re-embedded into a prearranged position in fresh paraffin blocks. The TMA blocks were heated at 45 °C for 1 h, sectioned at 4 μm, mounted onto slides and stored at -80 °C.

### Antibodies

The TMAs were analyzed using GeoMx Digital Spatial Profiling (DSP, Nanostring, Seattle, WA, USA). Four-color immunofluorescence was used for visualization of cell nuclei (Syto13), tumor cells (PanCk), immune cells (CD45) and T-cells (CD8) using the GeoMx Solid Tumor TME Morphology Kit v1.0 (Nanostring) combined with CD8 clone OTI3H6 (OriGene) preconjugated to Alexa Fluor 647 (2 µg/mL). Regions of Interest (ROIs) were profiled using 78 antibodies (Supplementary Table 1) consisting of the GeoMx panels for immune cell profiling (18 targets and 6 controls), immuno-oncology drug targets (10 targets), immune activation status (8 targets), cell death (10 targets), PI3K/AKT signaling (9 targets), immune cell typing (7 targets) and MAPK signaling (10 targets). The controls included positive housekeeper controls Histone H3, S6, and GAPDH and negative isotype controls mouse IgG1, mouse IgG2a and rabbit IgG.

### Sample preparation

The GeoMx Protein Slide Prep Kit for FFPE was applied with the GeoMx Protein Slide Preparation protocol (Nanostring) for all 12 slides in one processing batch. In brief, the TMA slides were baked at 60 °C followed by deparaffinization and rehydration (3 × 5 min Histolab-Clear (Histolab Product AB), 2 × 5 min 100% EtOH, 2 × 5 min 95% EtOH, 2 × 5 min ddH2O); antigen retrieval for 15 min in 1xCitrate buffer, pH 6.0 at high pressure, high temperature; and washing for 5 min in 1xTBS-T. A closed barrier on the slides was created by a hydrophobic pen. All the incubations were performed in a black humidity chamber. Slides were blocked (Buffer W, 1 h, RT); incubated with antibody cocktail diluted in Buffer W (overnight, 4 °C); washed in 1xTBS-T (3 × 10 min); fixed with 4% PFA (30 min, RT); washed in 1xTBS-T (2 × 5 min). Lastly, slides were stained with Syto13 (500 nM in TBS, 15 min RT), and dip-washed twice in 1xTBS-T.

### Sample processing

The TMA slides were scanned and processed in the GeoMx instrument. Immediately after scanning, 1–3 ROIs were defined per tumor using the polygon tool. ROIs were annotated as tumor or stroma depending on spatial localization. Each ROI was sequentially exposed to UV-light cleaving off the oligos coupled to the antibodies, before aspirating the oligos into separate wells in GeoMx collection 96-well plates. Aspirates were dried at 65 °C in a thermal cycler, rehydrated in 7 µL DEPC water and spun down. Probes were hybridized with GeoMx hyb codes corresponding to the GeoMx DSP Protein nCounter Readout scheme, incubated overnight at 67 °C and stored at 4 °C. Hybridized products were pooled by columns into strip tubes, in volumes related to the total segment area collected according to the GeoMx protocol. Quantification was performed using the nCounter (Nanostring) system, followed by transfer of readout data back to the GeoMx instrument.

### Construction of interactive software applications for GeoMx DSP data analysis

Four software applications were built in R shiny for interactive processing and analysis of data in the GeoMx DSP format: *app_normalization* for evaluation of normalization methods, *app_lmem* for linear mixed effect regression modelling (lmm) of data, *app_survival* for survival analysis, and *app_ml* for machine learning (ml) analysis. All applications were designed for compatibility with the GeoMx DSP data format and for ease of use in terms of data filtering, subsetting and adaptation of thresholds, and to handle outputs from the GeoMx analysis suite as well as NormalizerDE [27] for non-linearly scaled data. Features of the applications are described in Supplementary Fig. 1. Plots were mainly constructed using *ggplot2 (3.5.2)* and correlation matrices were based on *corrplot (0.92).* Lmm was implemented to account for multiple sampling per patient, using *lmerTest (3.1-3)* with the *lmerTest* function used in the univariate formula (Eq. (1)), making it a random intercept model with analysis executed for every biomarker and extracting p-value and coefficient from the results. P-values are provided in the *lmerTest* by using the t-value and degrees of freedom calculated via Satterwhaite’s method in a Student’s t-distribution. After execution for all biomarkers, p-values are false discovery rate (FDR) adjusted using the Benjamini-Hochberg method.

1 $${\rm{Expression}} \sim {\rm{Parameter + }}\left( {{\rm{1 | Patient ID}}} \right)$$

The *app_survival* includes both cox mixed effect (coxme) regression and Kaplan Meier analysis. The coxme model is from the *coxme (2.2–22)* package and handles one or more random effects, one always being Patient ID (Eq. (2)). The regression is univariate, thus the model is applied iteratively with one biomarker or parameter as fixed effect. Standard settings are used, except for allowing a change of optimization method in CoxMe.control: optpar, where “Nelder-Mead” was added as an alternative to the default method: “BFGS”. Coefficients and p-values are extracted from the CoxMe-fit. Kaplan Meier plots are generated using *survminer (0.5.0)*, complete with logrank test and error intervals. Kaplan Meier plots can be generated based on one sample per patient selected randomly at seed.

2 $$\eqalign{& {\rm{Surv}}\left( {{\rm{time}},{\rm{event}}} \right) \sim {\rm{Fixedeffect + }} \cr & \left( {{\rm{1|PatientID}}} \right){\rm{ + }}\left( {{\rm{1|Randomeffect}}\left( {\rm{s}} \right)} \right) \cr} $$

The *app_ml* uses the *randomForest* package for random forest classification/regression for defining biomarkers that vary across multiple categorical sample groups. Of note, the model does not account for patient dependency/bias so for data with multiple ROIs per patient, its use is limited for data exploration using all or one random sample per patient.

All additional packages utilized are stated in the readme-file provided with the applications. The applications are currently suited for analysis of protein data with nCounter read-out only (not transcriptomics) and are constructed to work on datasets exported from the GeoMx analysis suite without need for reformatting. The apps can also handle data normalized in NormalyzerDE [27] as described in the associated readme-file. The applications are available at https://github.com/CancerTargetLab/apps_geomx.

### DSP data and image analysis

Nuclei scaling was performed within the GeoMx analysis suite (Nanostring) prior to data export. No systematic batch effects were observed (Supplementary Fig. 2). Normalization approaches were evaluated based on mainly distribution of negative control signal in relation to ROI area or nuclear count, and distribution and correlation of typical tumor, immune and stroma markers in relation to ROI type (tumor/stroma) (Supplementary Fig. 3). Normalization by Variance Stabilizing Normalization (vsn) (applied in the NormalyzerDE [27] software) was identified as the most robust approach. For differential analysis, lmm was used as described above. Kaplan Meier analysis was performed as described above but based on average expression of biomarkers per patient across ROIs. We applied our previously described image analysis workflow [26] and adapted it to include the CD8 channel. Cox proportional hazard (coxph) modelling was performed in R with the package *survminer (0.5.0)* on averaged expressions per patient. From the GeoMx software, ROI images and ROI reports (image of an ROI with the ROI outline included) were downloaded as .tiff files for pre-processing in FIJI. All channels of the ROI image were subjected to background subtraction of 100 pixels and overlayed with corresponding ROI (.roi) extracted by masking the corresponding ROI report. These images were then imported to QuPath where cells were segmented, classified and exported. Segmentation was done using StarDist pretrained model (dsb2018 heavy augment.pb) in QuPath. Classification was performed by training a random forest model, first for stroma and tumor (PanCk channel) and second for CD8 and CD45 channel, respectively. The class and coordinates of each cell were then exported from QuPath to Python as a data frame. In Python, networkX was used to create a network graph with cells as nodes connected with edges if within Euclidean distances set to 30, 40 or 50 pixels, respectively, corresponding to 12, 16 and 20 μm in GeoMx images. The selected cells were CD8, CD45 and PanCk (tumor), producing six graphs (CD8:Tumor at 12/16/20 µm and CD45:Tumor at 12/16/20 µm). Using networkX, group degree centrality (gdc), representing fraction of tumor cells connected to at least one immune cell, was calculated from these graphs. The gdc metrics based on 30 pixels (12 μm) were selected for downstream analysis. Maximally selected rank statistics (*maxstat*,* 0.7.26)* was used to define a threshold for high/low gdc based on CD8 metrics and tumor ROIs.

### Data availability

The GeoMx data generated in this study are available in Zenodo, at DOI: 10.5281/zenodo.17395924.

## Results

### Study design and data preprocessing

**Fig. 1:**
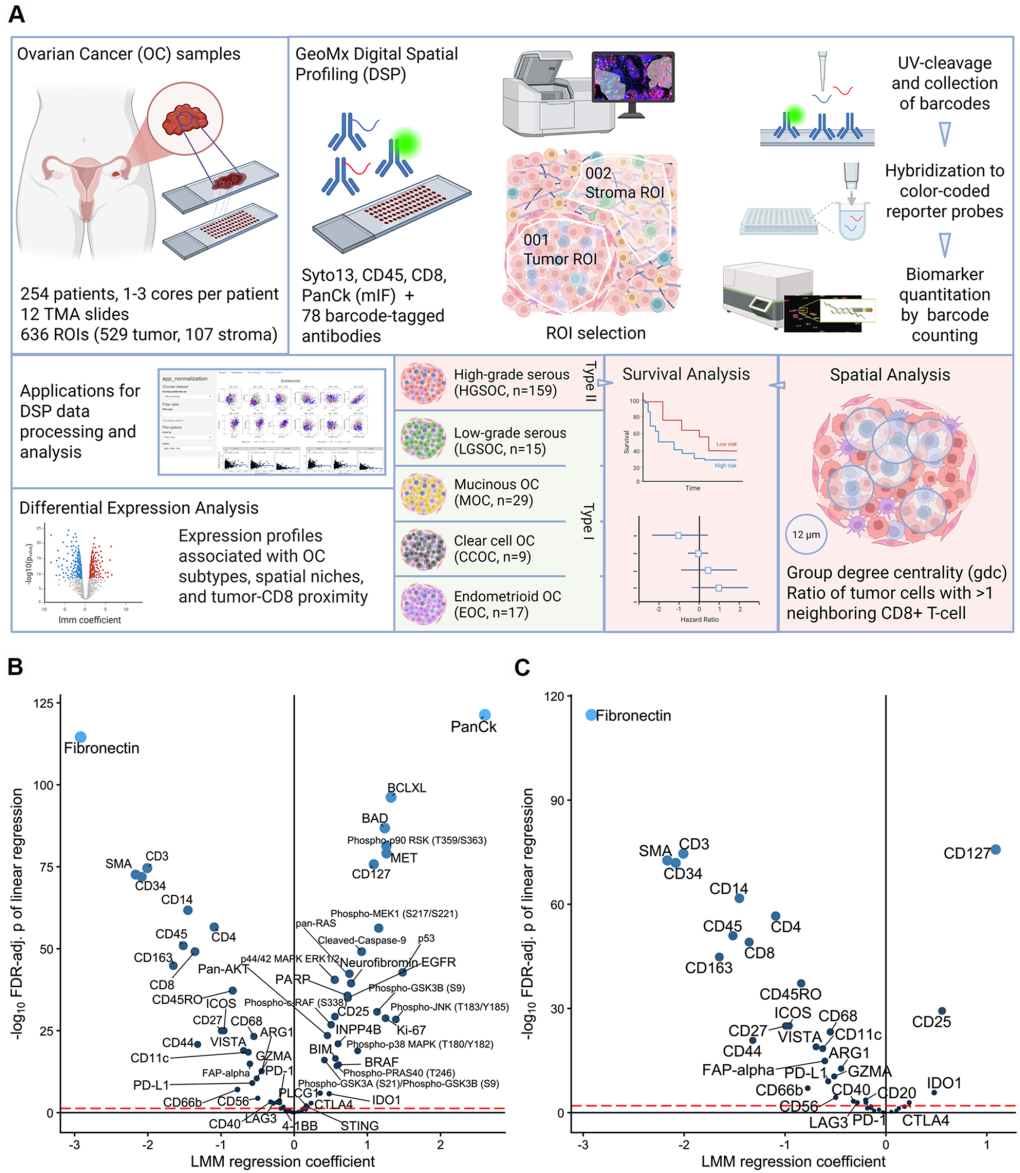
Study overview and data distribution. (**A**) Samples were collected from resected ovarian tissues. FFPE cores were distributed on 12 tissue microarray (TMA) slides. GeoMx DSP analysis involved multiplexed immunofluorescence (mIF) staining for CD45, CD8, PanCk and nuclei in addition to 78 non-visible, barcoded antibodies. Regions of interest (ROIs) were selected in tumor and stroma regions, respectively. Barcodes were released by ROI-specific UV exposure, followed by hybridization to reporter probes and quantification. Four R-shiny applications were developed for data processing and analysis. The purpose was characterization of tumor microenvironments of OC subtypes, and in the larger subgroup of HGSOC also in relation to prognosis and CD8-tumor spatial organization. Figure was generated in BioRender. (**B**) Tumor vs. stroma ROIs including all samples and all markers, and (**C**) immune/stroma-related markers only. Negative coefficients (left side of plot) = higher in stroma; positive coefficients (right side of plot) = higher in tumor. Analysis was based on linear mixed models (lmm) with Patient ID as random effect. Biomarkers with FDR-adjusted p-value < 0.05 (red dashed line) are displayed. Increased spot size and lighter shade indicate lower p-value.

In this study, digital spatial profiling was combined with computational image analysis to define TME profiles associated with OC histotypes and prognosis (Fig. 1A). TMAs were stained for PanCk, CD45, CD8 and Syto13 to guide ROI selection. A total of 636 ROIs were selected in tumor (*n* = 529) and tumor-adjacent stroma (*n* = 107) areas in tissue cores from 254 patients. The final dataset (Table 1) consisted of 192 malignant, 31 borderline, and 14 benign tumors, diagnosed as HGSOC (*n* = 159), MOC (*n* = 29), EOC (*n* = 17), LGSOC (*n* = 15), CCOC (*n* = 9), non-epithelial (*n* = 4), Brenner (*n* = 1, borderline), and teratoma (*n* = 1). In addition, 17 metastases that had spread to the ovaries from different sites were included. 78 biomarkers (Supplementary Table 1) were profiled in each ROI. For analysis, biomarkers were stratified into tumor- and immune/stroma-related markers, respectively, based on primary association.

**Table 1:** Clinical cohort

**Type I/II (** ***n*** **)**						
	Type I				Type II	NA
	70				159	25
**Histotype (n)**						
	EOC	LGSOC	MOC	CCOC	HGSOC	Other*
	17	15	29	9	159	25
**Malignant/Borderline/Benign (n)**						
Malignant	16	15	16	9	133	3
Borderline	1	0	7	0	21	2
Benign	0	0	6	0	5	3
Metastasis	0	0	0	0	0	17
**Stage (n)**						
I	11	3	14	4	16	2
II	0	2	1	0	9	0
III	3	10	1	5	91	0
IV	1	0	0	0	15	1
NA**	2	0	13	0	28	22
**Overall survival (years)**						
Average	7.8	6.5	9.1	3.5	5.1	5.2
Range	2.7–16.6	1.5–16.3	0.5–17.0	0.0-6.9	0.1–17.2	5.0-5.2
**ROIs collected (n)**						
Tumor areas	39	31	46	18	356	39
Stroma areas	8	10	8	5	73	3

*Samples not categorized as Type I/II OC, including metastases that had spread to the ovary, and primary non-epithelial, Brenner, and teratoma. **Includes metastases, benign and borderline

### CTLA-4 and IDO1 have higher expression in the tumor niche while PD-L1 and other immune-regulatory targets are more abundant in stroma

Analysis of tumor vs. stroma ROIs in the full cohort showed that the majority (82%) of the biomarkers had significant spatially differential expression which followed an expected pattern (e.g., higher Fibronectin, SMA, CD34 etc. in stroma; and higher PanCk, p53, Ki67, etc. in tumor) (Fig. 1B). The spatial distribution of specifically immune-related markers showed that e.g., CD25, CD127, and CTLA-4 were significantly higher in tumor compared to stroma regions (Fig. 1C), indicating that activated, immune regulatory T-cells are to a higher extent infiltrating the tumor niche than the stroma in OC tumors in general. The majority of immune markers were more abundant in stroma, most prominently T-cell markers (CD3, CD4 and CD8) but also myeloid (CD14, CD68, CD163, CD11c, and CD66b), B-cell (CD20), and NK-cell (CD56) markers. PD-L1 was also significantly higher in stroma compared to tumor, indicating that for OC in general, stromal cells, e.g., macrophages, are the main expressors of PD-L1. Macrophage-associated immune modulatory targets including ARG1 and VISTA were also significantly higher in stroma, whereas IDO1 was more highly expressed in the tumor niche, suggesting that tumor cells are the main contributors to IDO1 expression.

### Spatial signatures in Type I and Type II OC demonstrate a more active immune response in HGSOC and potential for differential immunotherapeutic targeting

**Fig. 2:**
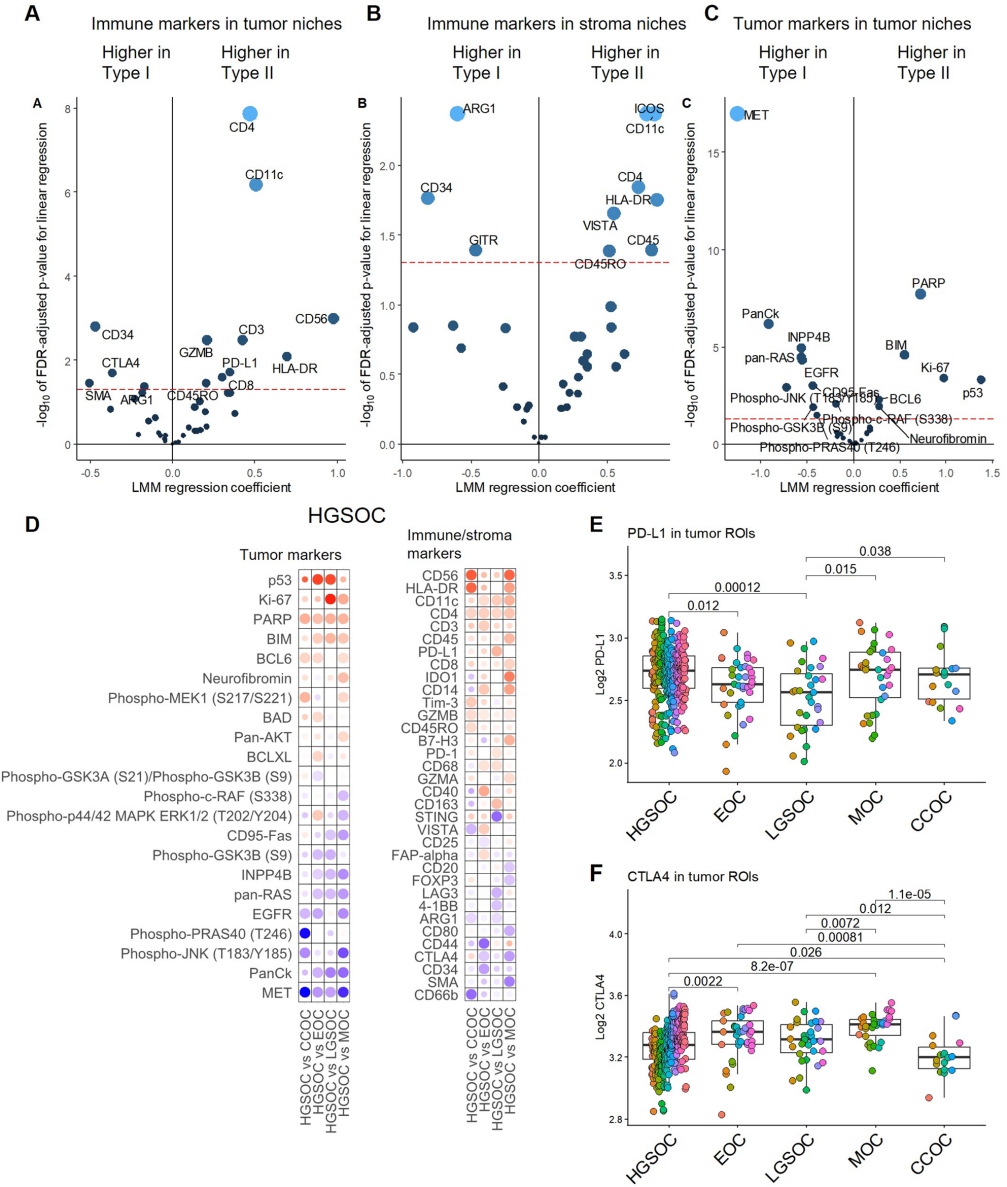
HGSOC Expression profiles and distribution of immune related targets in subtypes of OC. Differential expression in Type II vs. Type I OC (malignant samples only), displaying (A) immune markers measured in tumor regions; (B) immune markers measured in stroma regions; and (C) tumor markers measured in tumor regions. Negative coefficients (left side of plot) = higher in Type I; positive coefficients (right side of plot) = higher in Type II. Analysis is based on lmm models with Patient ID as random effect. Biomarkers with FDR-adjusted p-value < 0.05 (red dashed line) are displayed. Increased spot size and lighter blue color indicate lower p-value. (D) Intra-tumoral tumor (left) and immune (right) profiles of Type II HGSOC compared with other individual histotypes. Analysis was based on linear mixed models (lmm) with Patient ID as random effect. Biomarkers with p < 0.05 in at least one comparison are shown. Dots are colored by lmm coefficient and sized by significance. Red indicates higher and blue lower expression in HGSOC. Distribution of key immune-oncology targets (E) PD-L1 and (F) CTLA-4 expression across histotypes. Plots are based on malignant samples tumor ROIs and colored by Patient ID. Wilcoxon p-values are displayed.

Differential profiling of Type I and Type II OC showed higher intra-tumoral markers associated with antigen presentation (HLA-DR, CD11c), and T- and NK-cell infiltration (CD56, CD3, CD4, CD8, GZMB, CD45RO) in Type II OC, demonstrating that Type II tumors, which in this cohort were exclusively HGSOC, have stronger cytotoxic immune response from both T- and NK-cells, as well as higher presence of memory cells and antigen-presenting cells inside the tumor niche (Fig. 2A) compared to Type I tumors in general, as well as the individual Type I subtypes (Fig. 2D). Among established immunotherapeutic targets, PD-L1 showed significantly higher expression in Type II tumor niches (FDR adjusted *p* = 0.02, Fig. 2A). When stratified into histotypes, PD-L1 was at similar levels in HGSOC, MOC and CCOC, while lower in EOC and LGSOC, although expression levels varied extensively also within the individual subtypes (Fig. 2E). In contrast, CTLA-4 was more abundant in Type I tumor niches (FDR adjusted *p* = 0.02, Fig. 2A), particularly in MOC (Fig. 2F).

Differential profiling of tumor-adjacent stroma niches showed that CD11c, HLA-DR, CD4, CD45, CD45RO, ICOS and VISTA were higher expressed in Type II stroma, suggesting more activity of T-helper cells, co-stimulation and antigen presentation also in Type II stromal compartments compared to Type I stroma (Fig. 2B). In contrast, Type I stroma had significantly higher abundance of primarily ARG1, known to be expressed by TAMs and myeloid-derived suppressor cells (MDSCs) and suppressing T-cell function.

Tumor marker signatures showed that cell cycle related proteins were more abundant in Type II (Fig. 2C). Significantly higher expression of Ki67, p53, PARP, BCL6, and BIM reflect higher proliferation rate and known mutations in Type II OC, while PI3K and MAPK signaling pathways were dominating Type I OC, with particularly high MET expression.

### LGSOC exhibits high expression of STING and markers of apoptosis

**Fig. 3:**
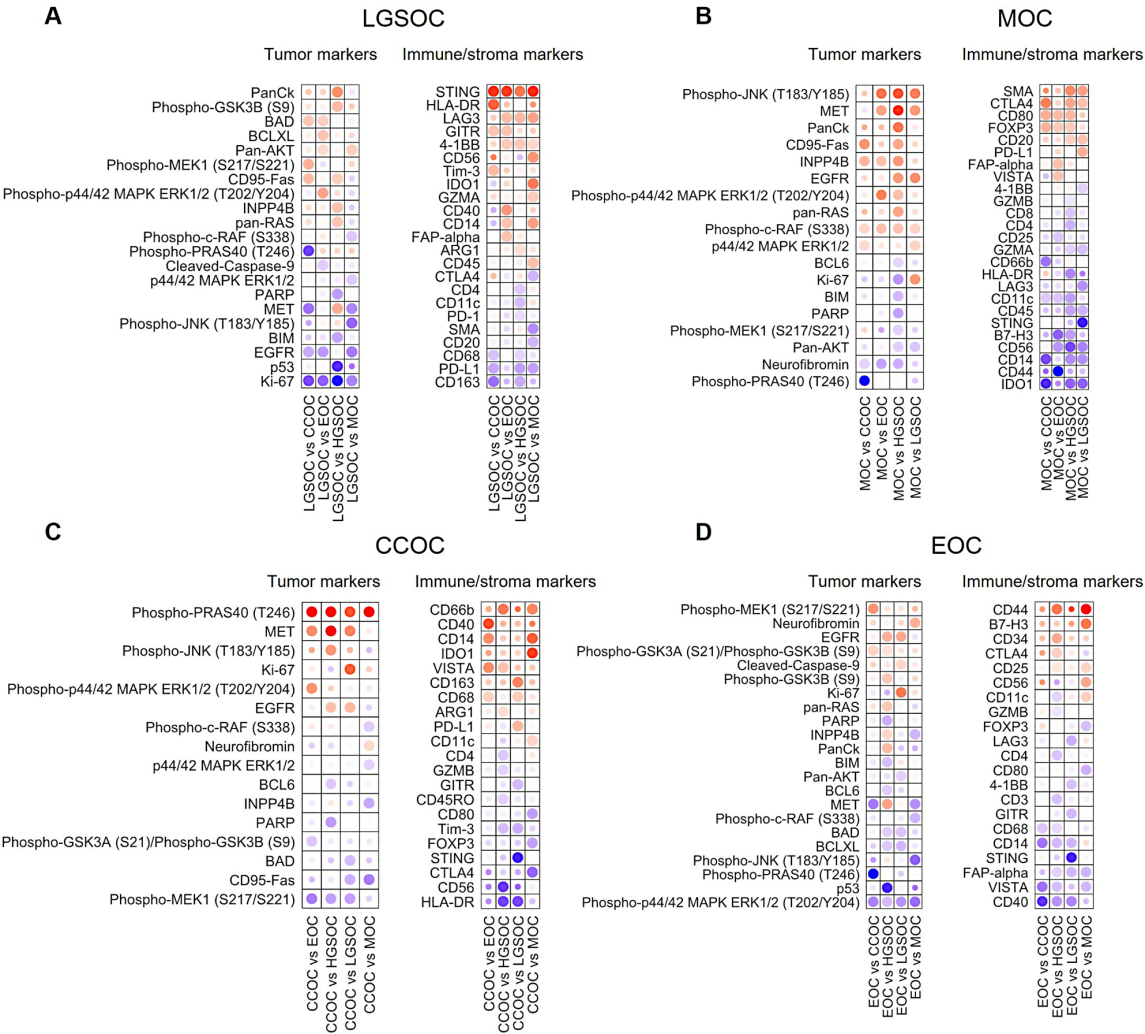
Intra-tumoral immune profiles of Type I OC histotypes. Each subfigure displays tumor (left) and immune/stroma (right) related biomarkers for (A) Low-grade serous OC (LGSOC), (B) Mucinous OC (MOC), (C) Clear cell OC (CCOC), and (D) Endometrioid OC, vs. other histotypes, including HGSOC. Analysis was based on linear mixed models (lmm) with Patient ID as random effect. Biomarkers with p < 0.05 in at least one comparison are shown. Dots are colored by lmm coefficient and sized by significance. Red indicates higher and blue lower expression in the histotype in focus.

The significantly lower PD-L1 expression in Type I OC was driven primarily by LGSOC, which was lower in PD-L1 compared to all other histotypes (Figs. 2E and 3A). In contrast, LGSOC had significantly higher expression of STING relative the other histotypes (Fig. 3A, Supplementary Fig. 4), which was also shown by us in a previous study [26] and may suggest a stronger potential for agonistic STING targeting rather than PD-1/PD-L1 blockade in LGSOC. STING activation leads to the production of type I interferons which promotes tumor antigen presentation and facilities T cell-mediated cytotoxicity [28]. In line with this, apoptosis associated tumor markers, including BCLXL, BAD and CD95-Fas, were also relatively high in LGSOC (Fig. 3A). Other immuno-oncology targets comparably abundant in LGSOC were LAG3, GITR and 4-1BB.

### MOC shows strong MAPK signalling and high PD-L1 and CTLA-4 expression

MOC displayed the overall highest intra-tumoral expression of PD-L1 across histotypes (Figs. 2E and 3B), as well as high intra-tumoral presence of Tregs (FoxP3, CTLA-4, CD80), and B-cells (CD20) (Figs. 2F and 3B). In addition, MOC was high in SMA and FAP-alpha, indicating a higher level of activated fibroblasts compared to other OCs. MOC also displayed high expression of MET, phospho-JNK, EGFR, CD95-Fas, INPP4B, p42/44 MAPK ERK1/2 (phosphorylated and unphosphorylated), pan-RAS, and phospho-c-RAF (Fig. 3B), indicative of strong MAPK signaling activity which is a known feature of MOC [29]. MAPK signaling has recently been demonstrated as associated with improved response to ICI in other tumors [30, 31], and our data thus indicate potential both for checkpoint (PD-1/PD-L1 and/or CTLA-4) and MAPK targeting of MOC.

### CCOC displays high infiltration of immune suppressive myeloid phenotypes and potential for IDO1, VISTA, MET or phospho-PRAS40 targeting

CCOC exhibited a vastly different immune profile compared to other histotypes, with not only stromal presence, but also markedly higher infiltration of macrophages and neutrophils, as indicated by high intra-tumoral CD66b, CD14, CD163, and CD68 and CD40 signals (Fig. 3C). Noteworthy, HLA-DR was low in CCOC, which could suggest that the myeloid cells in CCOC are a primarily suppressive and not associated with antigen presentation, potentially including MDSCs. Immune-suppressive markers IDO1, VISTA, and ARG1, which can be expressed by TAMs, were higher in CCOC relative to other histotypes, indicating potential for therapeutic targeting, particularly for subpopulations of CCOC patients displaying high intra-tumoral IDO1 expression (Supplementary Fig. 4). The strong immunosuppressive microenvironment can be related to significant expression of MET and phospho-PRAS40 (Fig. 3C) which was observed in CCOC. MET amplification triggers growth and angiogenesis and has previously been linked to immune suppression in ovarian cancer, with increased infiltration of e.g. MDSCs and Tregs [32]. Dysregulated phosphorylation of PRAS40, which affects the mTOR pathway, can also lead to an immunosuppressive microenvironment [33].

### EOC is characterized by low immune activation and high expression of B7-H3

EOC exhibited a less distinct immune profile, with overall low immune activation as shown by lower intra-tumoral levels of e.g. CD40, CD3, CD14 and CD68 (Fig. 3D). High expression of both CD44 and CD34 could indicate presence of hematopoietic stem cells in EOC. B7-H3, which has previously been associated with immune suppression and poor prognosis in OC [34], was strongly expressed in EOC (Supplementary Fig. 4), which motivates further studies to understand the potential for B7-H3 targeting to increase immune reactivity in EOC. EOC also had comparatively higher expression of phospho-MEK1, Neurofibromin, Cleaved Caspase-9, and EGFR, related to cell growth and apoptosis, while having lower expression of phospho-p44/42 MAPK ERK1/2 (Fig. 3D).

### Expression profiles associated with stage and malignant vs. benign/borderline reflect immune activation status

We compared early stage (I and II) to late stage (III and IV) tumors, as well as benign and borderline to malignant tumors to assess biomarker profiles related to tumor progression and malignant phenotype (Supplementary Table 2). Only HGSOC were subjected to analysis, as the rarer histotypes had too few patients and/or biased stage distribution (Table 1). Late stage HGSOC showed increased tumor proliferation (Ki67), T-cell infiltration (CD3), fibronectin and B7-H3 expression, compared to early stage, which were higher in e.g., p53, IDO1 and GZMA. Markers known to be associated with OC tumorigenesis such as neurofibromin, PARP, p53, BIM, cleaved caspase-9 and PLCG1, were higher in malignant tumors, while benign/borderline showed stronger MAPK and PI3K signaling activity. Overall T-cell presence as indicated by CD3, was higher in benign/borderline, but differential expression of CTLA-4 and CD80 are indicative of Tregs, in line with a stronger expression of CD4 and CD8 in malignant tumors. The strongest differentially expressed markers in benign/borderline were STING and beta-2-microglobulin, while e.g. PD-L1, B7-H3, CD11c, and CD40 were higher in malignant, reflecting early/late immune activity, respectively.

### Intra-tumoral CAF activation is strongly associated with poor prognosis in HGSOC

**Fig. 4:**
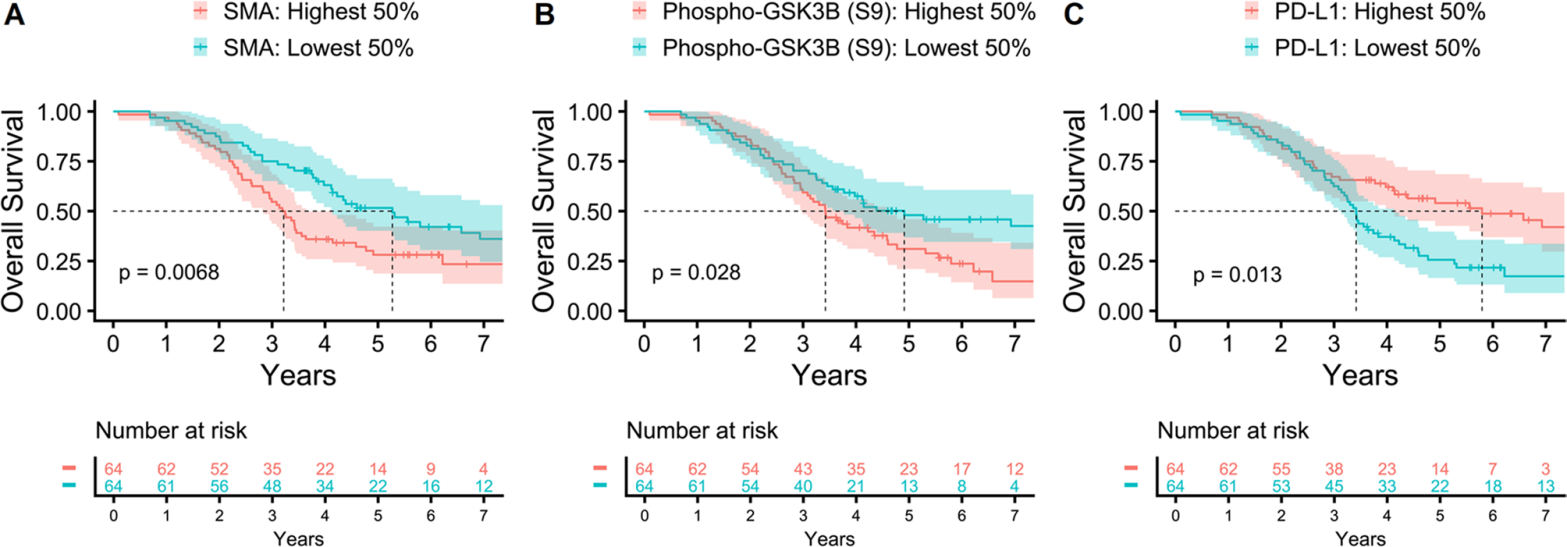
Prognostic markers in HGSOC. Kaplan-Meier analysis of overall survival of HGSOC patients using mean tumor ROI values per patient and dichotomization by 50th percentile for (**A**) SMA, p = 0.0068; (**B**) Phospho-GSK3B (S9), p = 0.028; and (**C**) PD-L1, p = 0.013. Dashed lines indicate median survival time.

Prognosis is inherently different for OC histotypes and, as expected, inferior survival was observed for HGSOC and CCOC patients in our data (Supplementary Fig. 5). HGSOC was the only subgroup large enough for robust survival analysis, where survival data was available from all but three patients. Three intra-tumoral markers were significantly associated with OS in Kaplan-Meier analysis (Fig. 4). SMA (Fig. 4A) and phospho-GSK3B (S9) (Fig. 4B) were associated with worse prognosis, suggesting that intra-epithelial fibroblast activation is a key prognostic marker in HGSOC, as phosphorylation of GSK3B at serine 9 is a key event in fibroblast function leading to increased SMA and contributing to fibrosis [35]. In contrast, PD-L1 (Fig. 4C) was associated with improved prognosis, consistent with marking immune infiltration and activity. In multivariate coxph models including significant clinical features (age and radical surgery), SMA and PD-L1 remained significant (SMA HR 1.25, *p* = 0.018; PD-L1 HR 0.75, *p* = 0.037) (Supplementary Fig. 6). While SMA was significantly higher in late-stage compared to early-stage HGSOC (Supplementary Fig. 7), the prognostic value of both SMA and PD-L1 were still significant in the late-stage subgroup on its own (Supplementary Fig. 8). For the three markers, optimally prognostic thresholds resulted in *p* = 0.00014 for SMA, *p* = 0.028 for phopho-GSK3B (S9), and *p* = 0.0076 for PD-L1, respectively (Supplementary Fig. 9).

### Integrated spatial and molecular analysis shows that CD8-to-tumor proximity is associated with prognosis and distinct tumor-immune molecular profiles

**Fig. 5:**
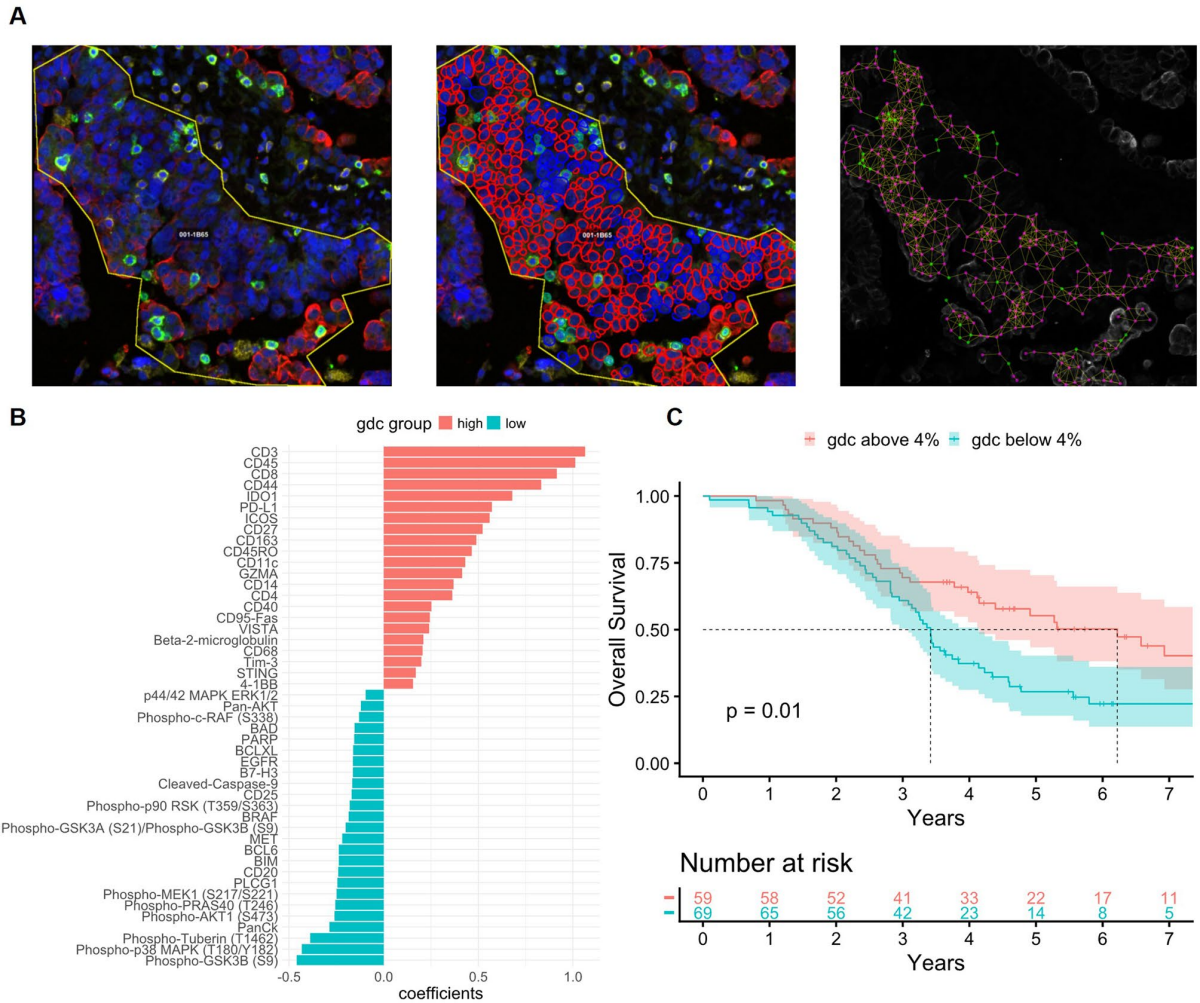
Spatial context of CD8 and tumor cell interaction and association to survival. (**A**) A representative ROI (220210-1B-65-001), in different stages of the image analysis. Left: the FIJI pre-processed ROI image (blue=DAPI, red=PanCk, green=CD45, yellow=CD8); Center: segmentation and cell classification in QuPath with cell outlines showing classes (red=tumor, green=CD45, yellow=CD8, blue=other); Right: the corresponding network graph produced in python and overlayed on the original ROI image with tumor cells represented by red and CD8^+^ cells by green nodes. (**B**) Differentially expressed proteins in the gdc-high and low groups based on optimal threshold: 4% of tumor cells within 12 µM of a CD8^+^ T cell. Analysis is based on lmm models with Patient ID as random effect, to account for varying numbers of ROIs per patient. Biomarkers with p-value < 0.05 are displayed. (**C**) Kaplan-Meier plot of survival of HGSOC patients dichotomized by optimal threshold. Dotted lines indicate median survival time

The spatial resolution of the GeoMx technology lies in the flexibility in defining precise regions for analysis; however, the spatial tissue architecture is not considered beyond annotations (here tumor or stroma) of each region. To assess the value of adding spatial statistics for stratification based on tumor-immune structure, we applied our GeoMx-compatible image analysis workflow [26]. Cells were assigned as nodes and cell-cell interaction within 30 pixels (12 μm) from each node as edges in network graph analysis for computation of spatial statistics (Fig. 5A). We assessed how gdc as a metric of spatial proximity between tumor and CD8 T-cells, was reflecting the overall molecular profile and prognosis of HGSOC. Linear mixed effect models with CD8-gdc dichotomized into high (> 4% of tumor cells connected to at least one CD8 + cell within 12 μm)) and low (< 4%) gdc in HGSOC, confirmed that CD8 gdc-high tumors had a significantly higher infiltration of both cytotoxic and helper T-cells, marked by higher CD8, CD3, CD45, CD4, CD44 and CD45RO (Fig. 5B). In addition, gdc-high tumors were higher in CD14, CD163, CD68, CD11c, and beta-2-microglobulin; hence close proximity between tumor cells and CD8 + cells in the tumor compartment implies increased infiltration also of myeloid cells, and higher MHCI expression. These tumors also showed higher expression of potential immuno-oncology targets such as IDO1, PD-L1, ICOS, VISTA, CD40, STING and Tim-3. In contrast, a more immune excluded microenvironment represented by the gdc-low tumors, was associated with significantly higher infiltration of B-cells (CD20) and potentially Tregs (CD25). CD8-gdc was also a prognostic marker, with gdc-high being associated with prolonged survival in HGSOC, both when dichotomized based on optimal threshold (Fig. 5C) and by 50th percentile (Supplementary Fig. 10A). The gdc-low group was also associated with significantly higher hazard ratio (1.78, *p* = 0.013) in a multivariate coxph model including significant clinical variables (Supplementary Fig. 10B). Of note, CD8 when measured on its own was not prognostic (*p* = 0.22, Supplementary Fig. 11), indicating that the tumor-CD8 proximity carries stronger prognostic value than CD8 without spatial context.

## Discussion

Ovarian cancers are largely treated the same, despite significant differences between histological subtypes and overall vast tumor heterogeneity. Immunotherapy has so far not shown much benefit in OC, even though a large fraction of tumors has significant immune infiltration [36, 37], which indicate potential for stratification by TME phenotypes. To contribute to the development of precision immuno-oncology for OC, we analyzed a large panel of key immune, stroma and tumor protein biomarkers to profile different OC histotypes by spatially resolved targeted proteomics. Our analysis particularly emphasizes the low-grade (Type I) OC histotypes for which tumor microenvironments have not previously been extensively characterized in one study. Although Type I OCs are generally considered indolent with longer survival compared to HGSOC [38], treatment options are highly limited for these patients, most of whom eventually succumb to the malignancy. Type I tumors are in general more genomically stable than HGSOC, resulting in poor response to various drugs, and generally lower immunogenicity. With the exception of CCOC, Type I tumors have therefore not been considered for ICI.

In light of emerging novel immunotherapeutic strategies, we show that distinct histotype-specific TMEs with expression of potential targets for immune stimulation can be identified. We noted that while intra-epithelial levels of phenotypic T-cell markers (CD3, CD8, CD4) were generally similar across the Type I histotypes, the T-cell functional markers indicative of activation status varied more. Importantly, the use of a high-plex antibody panel for dual immune and tumor profiling highlighted potential for combinatorial treatment. For example, MOC displayed strong MAPK signaling combined with high PD-L1 and CTLA-4 expression, while CCOC had significantly higher levels of MET and phospho-PRAS40 in combination with infiltration of macrophages and intra-epithelial expression of associated targets, including IDO1 and VISTA.

CCOC has inherently poor prognosis compared to other Type I OC tumors, which was also confirmed in our data. While PD-1 and CTLA-4 directed treatment has been considered for CCOC [17, 18], we show that both these targets, and particularly CTLA-4, are poorly expressed in the tumor niche of CCOC, which may explain the highly selective response to their treatment. CCOC is believed to arise from ovarian endometriosis [39], and the dysfunctional immune environment in endometriosis seems to persist after malignant transformation. Accordingly, our data showed that lymphocyte infiltration in general is low in CCOC, which is characterized by abundant macrophage infiltration and high expression of targets such as IDO1, CD40 and VISTA. For CCOC patients with poor response to conventional ICI, strategies for disrupting the TAM-driven immunosuppressive milieu may thus be more relevant. Tumor expression of particularly MET and phospho-PRAS40 was also highly significant in CCOC. Strong MET expression in CCOC has previously been reported [40, 41]. MET signaling in general has previously been linked to M2-like TAM polarization and immunosuppression, and MET inhibition may promote a more cytotoxic environment [42]. While little has been reported on the role of phospho-PRAS40 in CCOC, it is also a component of the PI3K/AKT signaling pathway and may be relevant to explore as a target for this histotype.

Like CCOC, EOC is also considered an endometriosis-associated cancer, but morphologically and biologically distinct from CCOC [43]. The microenvironment also differed, with EOC having overall low lymphoid and myeloid infiltration, consistent with strong expression of B7-H3, a potent inhibitor of anti-tumor immunity and associated with an immune cold environment [44]. B7-H3 could thus be investigated as a potential target for priming immune responses in EOC. MOC, in contrast, displayed a relatively high intra-tumoral expression of PD-L1 and CTLA-4, but also significant SMA and FAP-alpha expression associated with dense fibrosis which is a known feature of MOC tumors. Poor therapeutic response in MOC may thus be primarily caused by the stromal physical barriers, and combinatorial treatments directed against fibroblasts, e.g. targeting FAP or TGF-β [45, 46] or immune-modulating mucins [47] could potentially confer an improved efficacy.

LGSOC is now known to be histopathologically distinct from its HGSOC counterpart and arises from serous borderline tumors, rather than from serous tubal precursor lesions which is the origin of HGSOC [48]. The TME of LGSOC is generally considered immunosuppressive [49], which was also confirmed in our data, in which LGSOC displayed low intra-epithelial infiltration of both lymphoid and myeloid cells, and low expression of PD-L1 and PD-1. However, LGSOC also had high expression of HLA-DR, which was previously shown by us in a smaller cohort [26], and which may indicate immune reactivity. In addition, STING was significantly higher in LGSOC, as previously observed by us^26^ and others [50]. The cGAS-STING pathway is activated by presence of cytosolic DNA from pathogens, but also from cytosolic self-DNA which can accumulate due to cell stress and DNA damage in tumors [51]. Stimulation of the cGAS-STING pathway is thus an attractive option for priming anti-tumor immunity, which has been explored for different cancers, including ovarian [52, 53]. As shown by Bruand et al., STING stimulation may however have a reverse effect in BRCA mutated tumors [54], which indicates that agonistic STING treatment may be more beneficial in less inflamed, BRCA wildtype tumors. Whether the consistently high STING expression in the poorly immunogenic LGSOC tumors implies an opportunity for STING-directed immune priming remains to be elucidated.

While the data reveals general histotype-associated implications for therapeutic potential, histotyping will likely not be sufficient to inform clinical decision making. Given the heterogeneity and patient variation also within OC histotypes, diagnostics assays for stratification of tumors will be needed to predict treatment susceptibility. Such tools could be based on tissue image analysis from staining with condensed panels, including key markers as identified in the current study, for classification based on spatio-molecular TME profiles. Imaging by conventional multiplex immunofluorescence is less costly and complex compared to spatial omics analyses, and computational image analysis can detect infiltration patterns associated with TME expression profiles. In this study, we showcased this on HGSOC, by applying our previously established image analysis pipeline based on graph networks [26]. Group degree centrality as a spatial metric for tumor-CD8 interaction was shown to carry stronger prognostic value than CD8 expression on its own, demonstrating the added value of spatial context. Gdc-high tumors also showed association to expression of key immuno-oncology targets (PD-L1, IDO1, ICOS and more). In contrast, gdc-low (immune excluded) tumors showed significantly stronger PI3K/AKT signaling as well as CD20 and CD25 inside the tumor niche. While presence of tertiary lymphoid structures is associated with beneficial prognosis in OC [55], a more intra-epithelial and diffuse distribution of B-cells may thus indicate an immune suppressed milieu. Similar to the immune excluded HGSOCs, MOC also displayed high B-cell infiltration together with Tregs, again indicating that B-cells may deactivate the immune response when dispersed inside the tumor nests. The potential regulatory role of B-cells has also been demonstrated by others and discussed in relation to therapeutic implications [56, 57].

Like most studies on OC, and especially the rarer histotypes, the current study is limited by low patient numbers, particularly for CCOC (9 patients). Extensive quality control and expected differential signatures for tumor and stroma, and Type I/II tumors adds validity to the data, and many of the histotype-specific markers were identified also in a smaller cohort [26]. Still, the findings need validation in cohorts that preferably are sufficiently large to explore the prognostic association with marker expression also for the rarer OC subtypes, as well as functional analysis to investigate the potential for targeting. Analysis of TMA cores from heterogeneous OC tumors is another limitation, which here was addressed by selection of multiple ROIs in up to three cores per tumor and analysis using mixed effect models where patient ID was added as a random effect. In general, analysis of spatial omics data suffers from lack of standardized approaches and widely applicable bioinformatic tools. The softwares presented as part of this study have greatly improved usability and transparency of our in-house GeoMx data analysis workflow, and we hope that they will contribute to the wider user community as well.

In summary, we present tumor and microenvironment signatures associated with individual OC histotypes. We show that spatial tumor-CD8 interactions in HGSOC are more prognostic than CD8 expression on its own and marks presence of key immuno-oncology targets in the tumor niche. Importantly, our results highlight opportunities for immune priming also in poorly immunogenic tumors, including immune excluded HGSOC and the Type I OC histotypes. The data thus indicate potential for differential targeting related to both histotyping and spatial phenotyping in OC, which holds promise for improved personalized treatment of these complex cancers.

## Supplementary Information

Below is the link to the electronic supplementary material.

Supplementary Material 1

## Data Availability

Data are available in Zenodo, at DOI: 10.5281/zenodo.17395924. The applications developed for GeoMx data analysis are available at https://github.com/CancerTargetLab/apps_geomx.

## Abbreviations

OC: Ovarian carcinoma
PARP: Poly (ADP-ribose) polymerase
TME: Tumor microenvironment
Treg: Regulatory T-cells
TAM: Tumor associated macrophage
CAF: Cancer associated fibroblast
ICI: Immune checkpoint inhibition
HGSOC: High-grade serous ovarian carcinoma
LGSOC: Low-grade serous ovarian carcinoma
EOC: Endometrioid ovarian carcinoma
CCOC: Clear cell ovarian carcinoma
MOC: Mucinous ovarian carcinoma
DSP: Digital spatial profiling
TMA: Tissue microarray
FFPE: Formalin-fixed paraffin-embedded
ROI: Region of interest
lmm: linear mixed effect modelling
ml: Machine learning
FDR: False discovery rate
coxme: cox mixed effect
VSN: Variance stabilizing normalization
coxph: cox proportional hazard
gdc: group degree centrality
MDSC: Myeloid-derived suppressor cell
