# Supplementary material for: Distinct immune microenvironments in ovarian cancer subtypes indicate potential for immunotherapies
Source: J Transl Med. 2026 Feb 21;24:337. doi: 10.1186/s12967-026-07879-8 (PMC12973683; doi:10.1186/s12967-026-07879-8)
Supplement: Supplementary file 1 — Supplementary Material 1 [file 12967_2026_7879_MOESM1_ESM.pdf]

Supplementary Table 1. Biomarkers for tumor-immune profiling

Biomarker	Inclusion subpanel (tumor, immune or control)	GeoMx panel module
Beta-2-microglobulin	Tumor	Immune Cell Profiling Panel
CD11c	Immune	Immune Cell Profiling Panel
CD20	Immune	Immune Cell Profiling Panel
CD3	Immune	Immune Cell Profiling Panel
CD4	Immune	Immune Cell Profiling Panel
CD45	Immune	Immune Cell Profiling Panel
CD56	Immune	Immune Cell Profiling Panel
CD68	Immune	Immune Cell Profiling Panel
CD8	Immune	Immune Cell Profiling Panel
CTLA4	Immune	Immune Cell Profiling Panel
Fibronectin	Immune	Immune Cell Profiling Panel
GAPDH	Control	Immune Cell Profiling Panel
GZMB	Immune	Immune Cell Profiling Panel
Histone H3	Control	Immune Cell Profiling Panel
HLA-DR	Immune	Immune Cell Profiling Panel
Ki-67	Tumor	Immune Cell Profiling Panel
Ms IgG1	Control	Immune Cell Profiling Panel
Ms IgG2a	Control	Immune Cell Profiling Panel
PanCk	Tumor	Immune Cell Profiling Panel
PD-1	Immune	Immune Cell Profiling Panel
PD-L1	Immune	Immune Cell Profiling Panel
Rb IgG	Control	Immune Cell Profiling Panel
S6	Control	Immune Cell Profiling Panel
SMA	Immune	Immune Cell Profiling Panel
4-1BB	Immune	IO Drug Target Panel
ARG1	Immune	IO Drug Target Panel
B7-H3	Immune	IO Drug Target Panel
GITR	Immune	IO Drug Target Panel
IDO1	Immune	IO Drug Target Panel
LAG3	Immune	IO Drug Target Panel
OX40L	Immune	IO Drug Target Panel
STING	Immune	IO Drug Target Panel
Tim-3	Immune	IO Drug Target Panel
VISTA	Immune	IO Drug Target Panel
CD127	Immune	Immune Activation Status Panel
CD25	Immune	Immune Activation Status Panel
CD27	Immune	Immune Activation Status Panel
CD40	Immune	Immune Activation Status Panel
CD44	Immune	Immune Activation Status Panel
CD80	Immune	Immune Activation Status Panel
ICOS	Immune	Immune Activation Status Panel
PD-L2	Immune	Immune Activation Status Panel
BAD	Tumor	Cell Death Panel
BCL6	Tumor	Cell Death Panel
BCLXL	Tumor	Cell Death Panel
BIM	Tumor	Cell Death Panel
CD95-Fas	Tumor	Cell Death Panel
Cleaved-Caspase-9	Tumor	Cell Death Panel
GZMA	Immune	Cell Death Panel
Neurofibromin	Tumor	Cell Death Panel
p53	Tumor	Cell Death Panel
PARP	Tumor	Cell Death Panel
INPP4B	Tumor	PI3K/AKT Signaling Panel
MET	Tumor	PI3K/AKT Signaling Panel
Pan-AKT	Tumor	PI3K/AKT Signaling Panel
Phospho-AKT1 (S473)	Tumor	PI3K/AKT Signaling Panel
Phospho-GSK3A (S21)/Phospho-GSK3B (S9)	Tumor	PI3K/AKT Signaling Panel
Phospho-GSK3B (S9)	Tumor	PI3K/AKT Signaling Panel
Phospho-PRAS40 (T246)	Tumor	PI3K/AKT Signaling Panel
Phospho-Tuberin (T1462)	Tumor	PI3K/AKT Signaling Panel
PLCG1	Tumor	PI3K/AKT Signaling Panel
CD14	Immune	Immune Cell Typing Panel
CD163	Immune	Immune Cell Typing Panel
CD34	Immune	Immune Cell Typing Panel
CD45RO	Immune	Immune Cell Typing Panel
CD66b	Immune	Immune Cell Typing Panel
FAP-alpha	Immune	Immune Cell Typing Panel
FOXP3	Immune	Immune Cell Typing Panel
BRAF	Tumor	MAPK Signaling Panel
EGFR	Tumor	MAPK Signaling Panel
p44/42 MAPK ERK1/2	Tumor	MAPK Signaling Panel
pan-RAS	Tumor	MAPK Signaling Panel
Phospho-c-RAF (S338)	Tumor	MAPK Signaling Panel
Phospho-JNK (T183/Y185)	Tumor	MAPK Signaling Panel
Phospho-MEK1 (S217/S221)	Tumor	MAPK Signaling Panel
Phospho-p38 MAPK (T180/Y182)	Tumor	MAPK Signaling Panel
Phospho-p44/42 MAPK ERK1/2 (T202/Y204)	Tumor	MAPK Signaling Panel
Phospho-p90 RSK (T359/S363)	Tumor	MAPK Signaling Panel

Supplementary Table 2. Significant (p<0.05) differentially expressed markers in A) early vs late stage HGSOC and B) benign/borderline vs malignant HGSOC.

A	Higher in early stage (I+II)	Higher in late stage (III+IV)
HGSOC (all markers)	P53, Phospho-p38 MAPK (T180/Y182), IDO1, GZMA, PanCk, CD27, GITR	Ki-67, Fibronectin, CD3, B7-H3, Beta-2-microglobulin

B	Higher in benign + borderline	Higher in malignant
HGSOC<br>Tumor-associated markers	Beta-2-microglobulin, pan-RAS, INPP4B, MET, Phospho-c-RAF (S338), Phospho-GSK3B (S9), Phospho-MEK1 (S217/221), CD95/FAS, Phospho-PRAS40 (T246)	Neurofibromin, PARP, p53, BIM, Cleaved Caspase 9, PLCG1
HGSOC Immune/stroma-associated markers	STING, LAG3, Fibronectin, GITR, 4-1BB, ARG1, CD3, CD80, CTLA4, FAP-alpha	HLA-DR, PD-L1, CD68, CD8, CD11c, B7-H3, CD4, CD163, SMA, CD40

Supplementary Figure 1

A

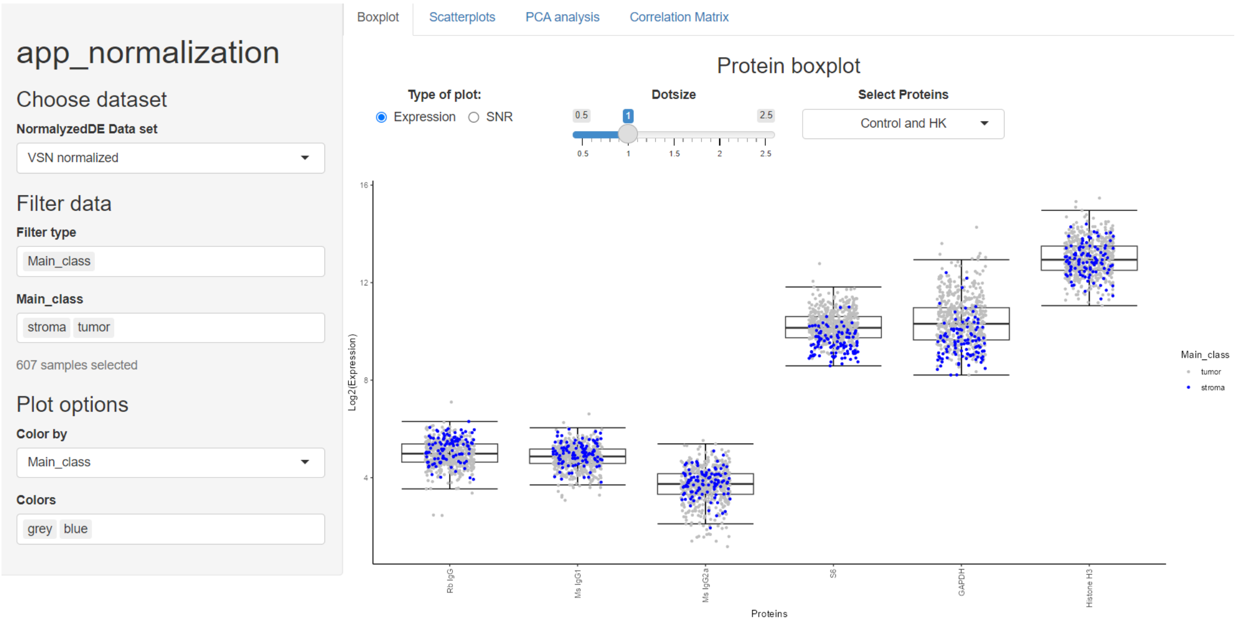

B

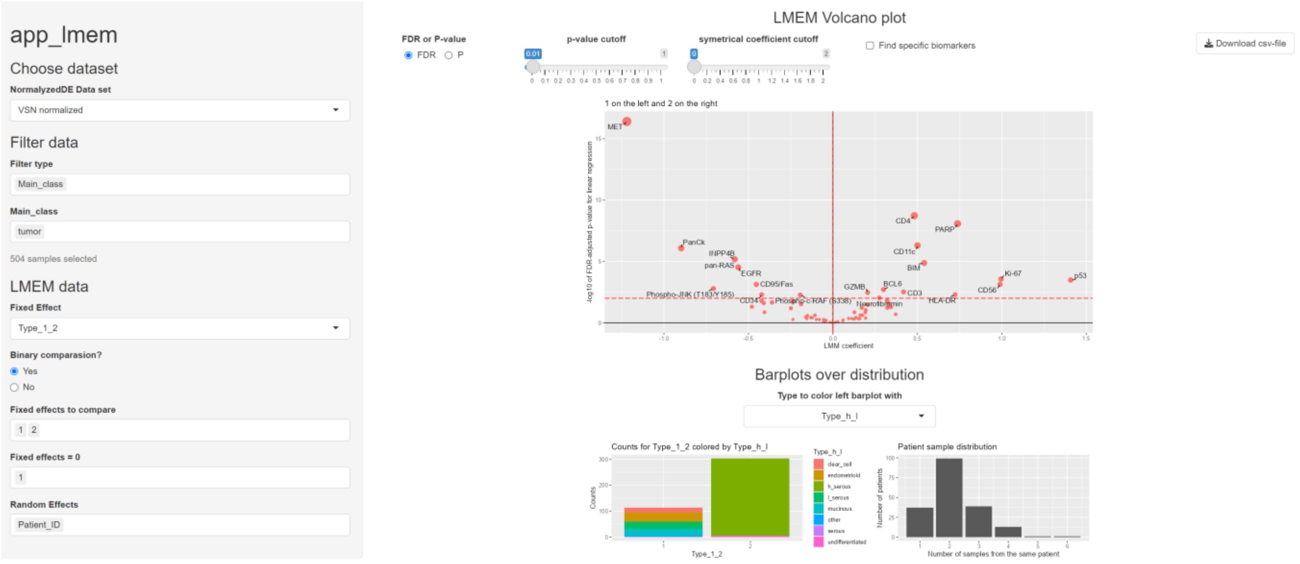

C

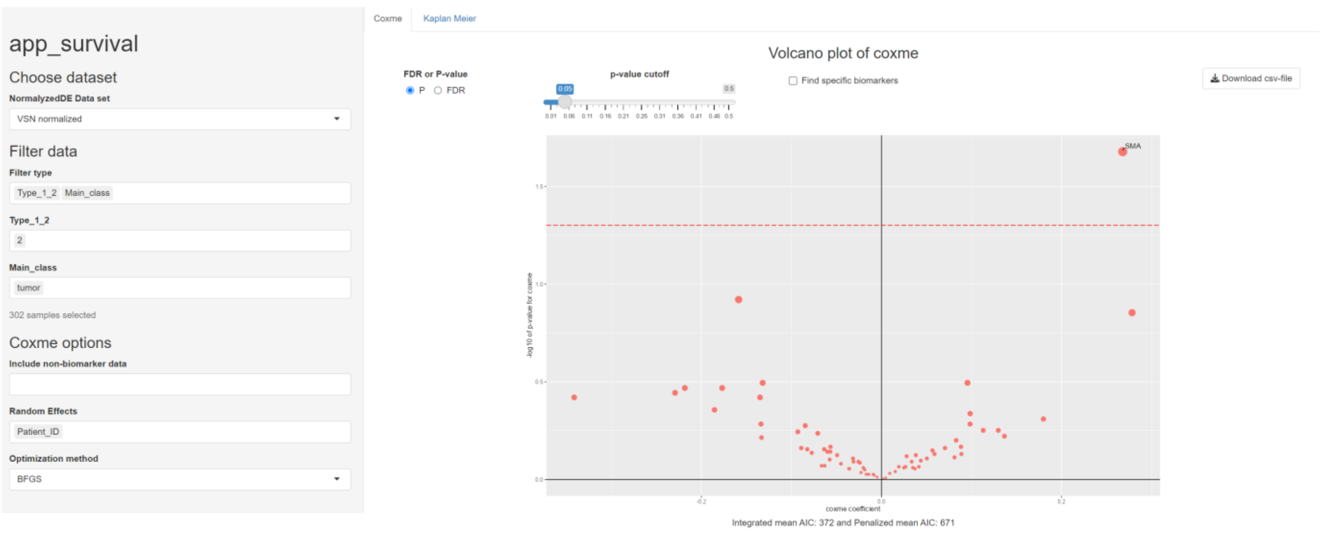

D

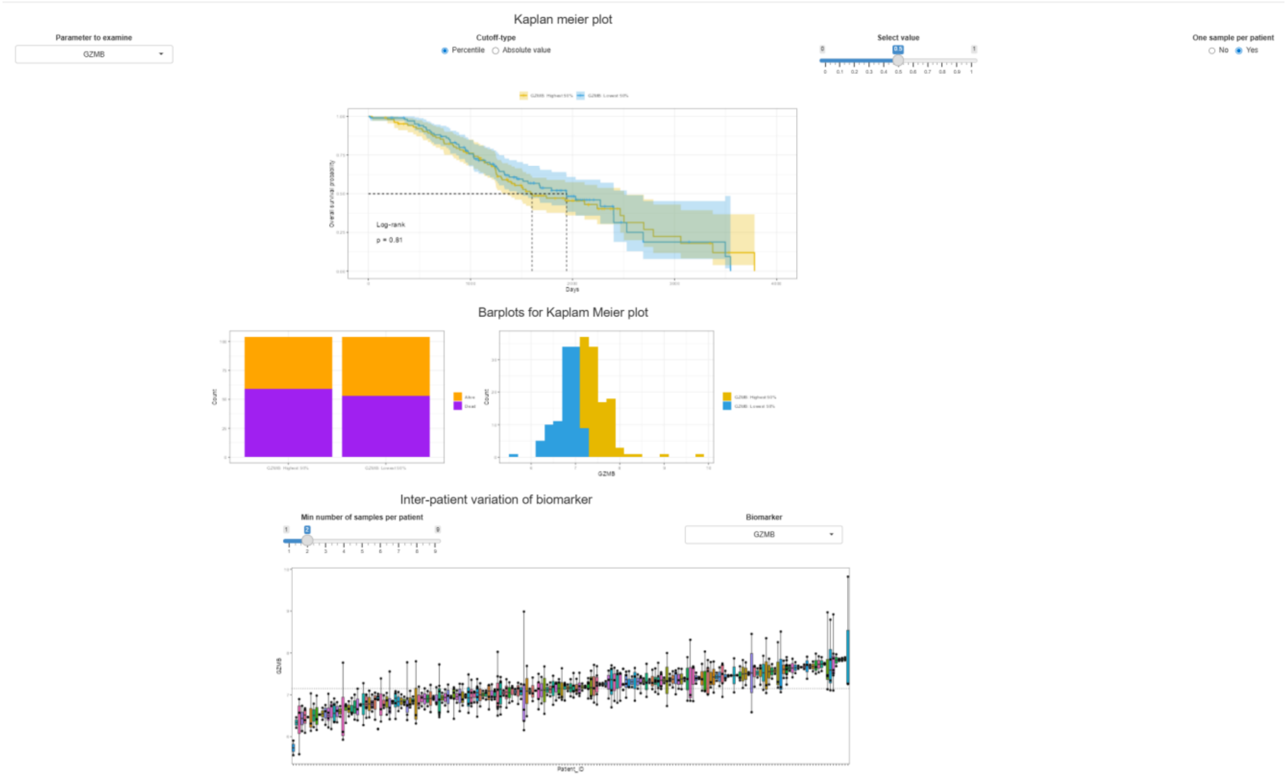
Kaplan meier plot
Parameter to examine
GZMB
Cutoff-type
Percentile
Absolute value
Select value
One sample per patient
No
Yes
Log-rank
p = 0.81
Days
Barplots for Kaplam Meier plot
GZMB
Inter-patient variation of biomarker
Min number of samples per patient
Biomarker
GZMB
Patient_ID

E

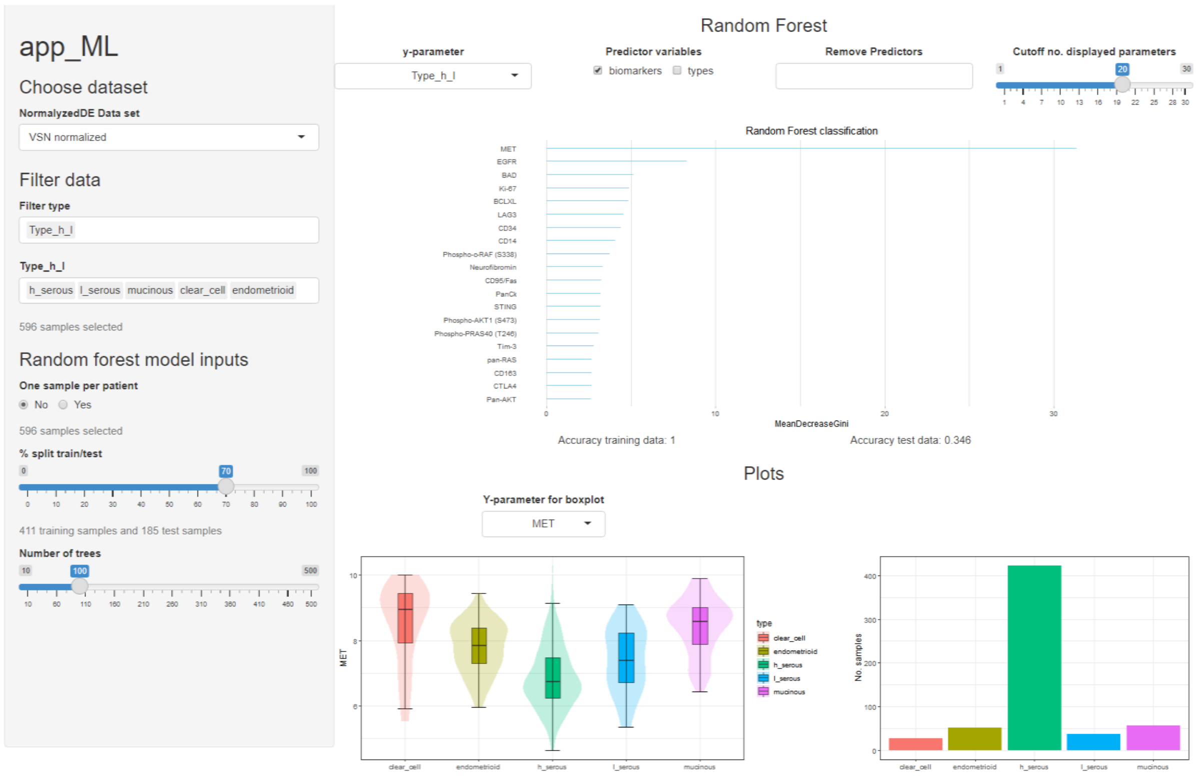
app_ML
Choose dataset
NormalizedDE Data set
VSN normalized
Filter data
Filter type
Type_h_l
Type_h_l
h_serous l_serous mucinous clear_cell endometrioid
596 samples selected
Random forest model inputs
One sample per patient
No
Yes
596 samples selected
% split train/test
411 training samples and 185 test samples
Number of trees
Random Forest
y-parameter
Type_h_l
Predictor variables
biomarkers
types
Remove Predictors
Cutoff no. displayed parameters
Random Forest classification
MET
EGFR
BAD
Ki-67
BCLXL
LAG3
CD34
CD14
Phospho-c-RAF (S338)
Neurofibromin
CD95/Fas
PanCk
STING
Phospho-AKT1 (S473)
Phospho-PRAS40 (T246)
Tim-3
pan-RAS
CD163
CTLA4
Pan-AKT
MeanDecreaseGini
Accuracy training data: 1
Accuracy test data: 0.346
Plots
Y-parameter for boxplot
MET
type
clear_cell
endometrioid
h_serous
l_serous
mucinous
No. samples

Supplementary Figure 1. Software and features.

A) Screenshot from *app_normalization.* The app is designed for comparison of marker distributions across e.g. sample subsets, to investigate different ways of normalizing data in relation to the raw data. The sidebar has a similar layout for all tabs and applications. Here, the vsn normalized data has been selected in the dropdown menu, which in this case also include raw GeoMx data, and datasets normalized by CyclicLoess, and linear scaling by control markers. Any annotations from the GeoMx sample file can be used to filter data, and additional annotations can be added as an “extra data” file (in our case, this included e.g. the spatial metrics which were calculated after the GeoMx analysis). The main panel include several options for plot settings, including data to plot (expression or signal-to-noise; all proteins, control markers or selected proteins) and display settings. Here, the Boxplot tab is shown. The other tabs include scatterplots, PCA plots and correlation matrices.

B) Screenshot from *app_lmem.* Linear mixed effect models are used to identify biomarkers associated with binary or ordinal sample variables. Fixed and random effects are defined. The data is displayed in a volcano plot with –log p-value on y-axis and regression coefficient on the x-axis. Identity of biomarkers above the defined significance thresholds are marked, or specific biomarkers as defined in a dropdown menu can be marked. The results including p-value, FDR and lmm coefficients can be downloaded as a csv file. Distribution of samples are shown in barplots which can be colored by any annotation.

C) Screenshot from *app_survival.* The first tab is shown in which cox mixed effect regression is performed, outputting a volcano plot. Data is filtered similar to the other apps, but non-biomarker data (i.e., clinical variables or spatial metrics) can also be included.

D) In the second tab in *app_survival,* Kaplan-Meier plots of specific biomarkers or other variables can be generated. Different thresholds can be explored, and all or one sample per patient can be included. Corresponding data distribution by high/low groups are shown in a barplot and histogram, and distribution of biomarkers across patients is shown as boxplots.

E) Screenshot from *app_ML*. The machine learning application performs random forest analysis to explore biomarkers driving variation across multiple sample groups. All or one (randomly selected) ROI per patient can be used as model input. Thresholds for dividing data into train and test sets, as well as number of trees can be defined. Mean Decrease Gini is plotted to visualize variable impact on model performance. Specific variables or biomarkers can be visualized as boxplots across sample categories and sample distribution is shown in barplots.

Supplementary Figure 2

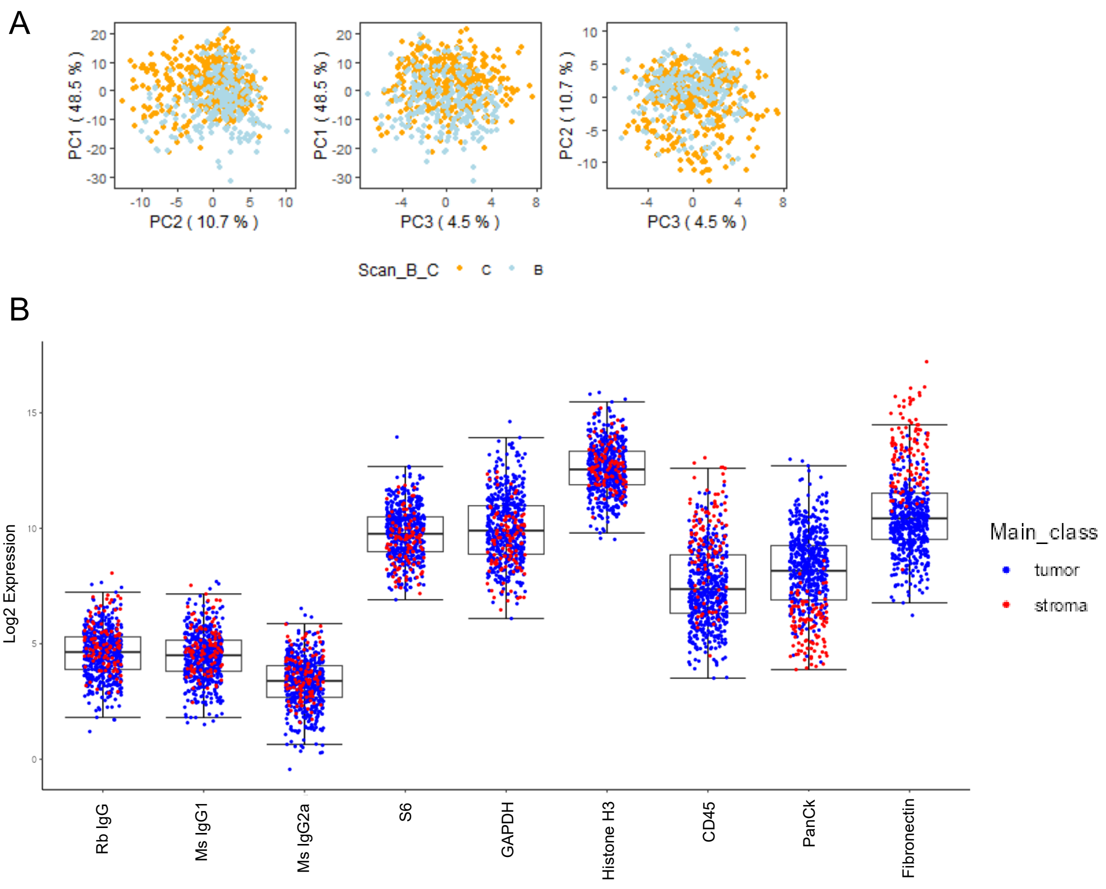

Supplementary Figure 2. Distribution of non-normalized data. A) PCA plots for the first three principal components, colored by sample batch where samples for TMA B (Scan B) was collected 2001-2010, and TMA C (Scan C) 2011-2015. B) Boxplots for the three negative (isotype) controls and positive (house-keeper) proteins showing similar distribution across ROI types (tumor and stroma). CD45, PanCk and Fibronectin are included as references, showing expected distribution in tumor/stroma.

Supplementary Figure 3

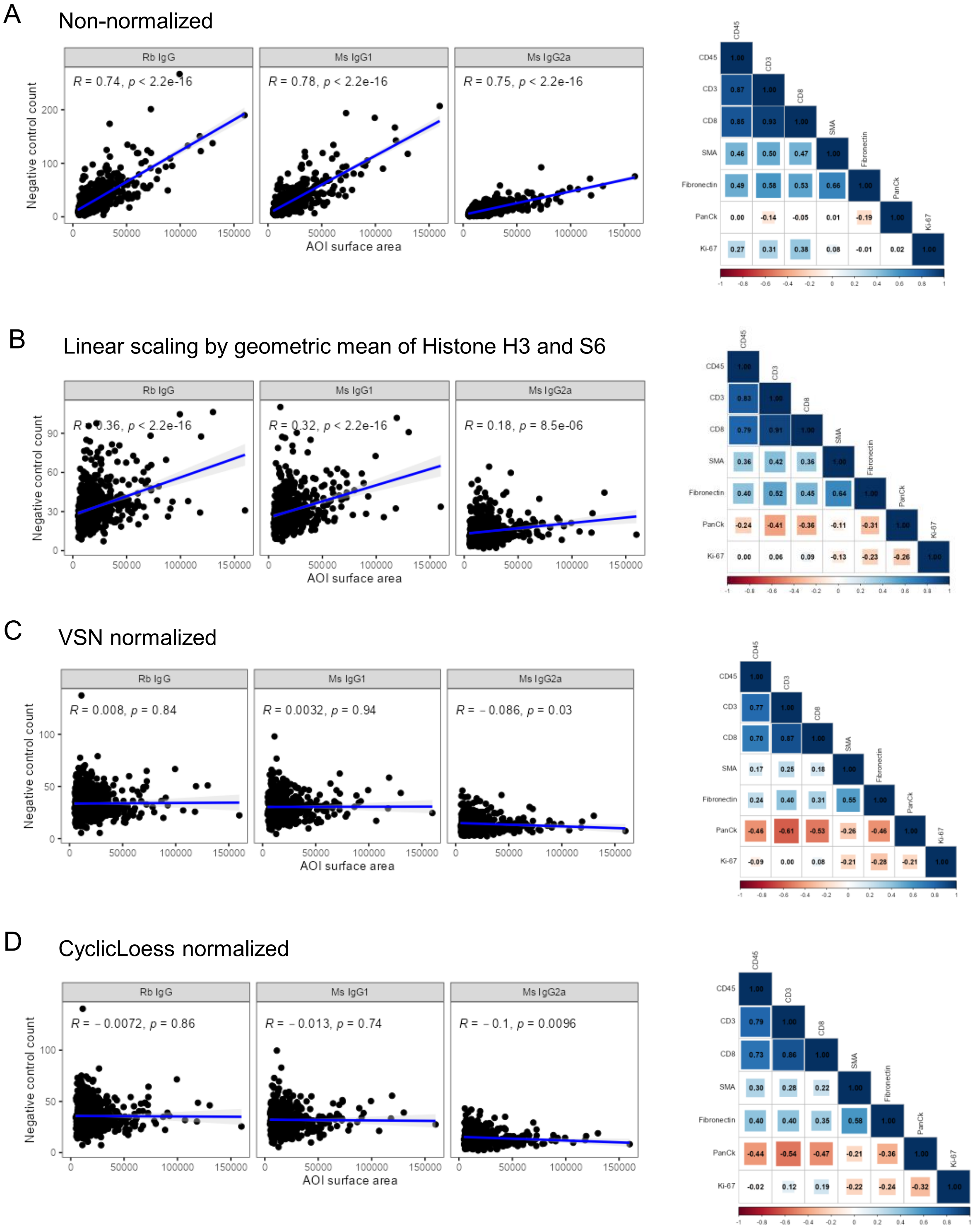

Supplementary Figure 3. Evaluation of normalization approaches. Left panel: Scatterplots of negative (isotype) controls to ROI area, including pearson-correlation coefficient and p-value. AOI = area of illumination, here the same as ROI as no ROI segmentation was performed prior to probe collection. Right panel: Correlation matrix of selected immune/stroma/tumor proteins with expected correlation patterns. The displayed datasets include A) non-normalized data; B) data linearly scaled by geometric mean of Histone H3 and S6 (following recommendations by Nanostring). GAPDH was not used as it showed poorer correlation to the other to house-keeper proteins; C) variance stabilizing normalization (vsn) – normalized data; and D) CyclicLoess normalized data. Vsn and CyclicLoess normalization was performed in the NormalizerDE software. Improved normalization was observed with non-linear methods (vsn and cyclicLoess) for addressing background signal variation by ROI size, as well as providing expected marker distribution pattern, e.g., negative correlation between PanCk and immune/stroma markers.

Supplementary Figure 4

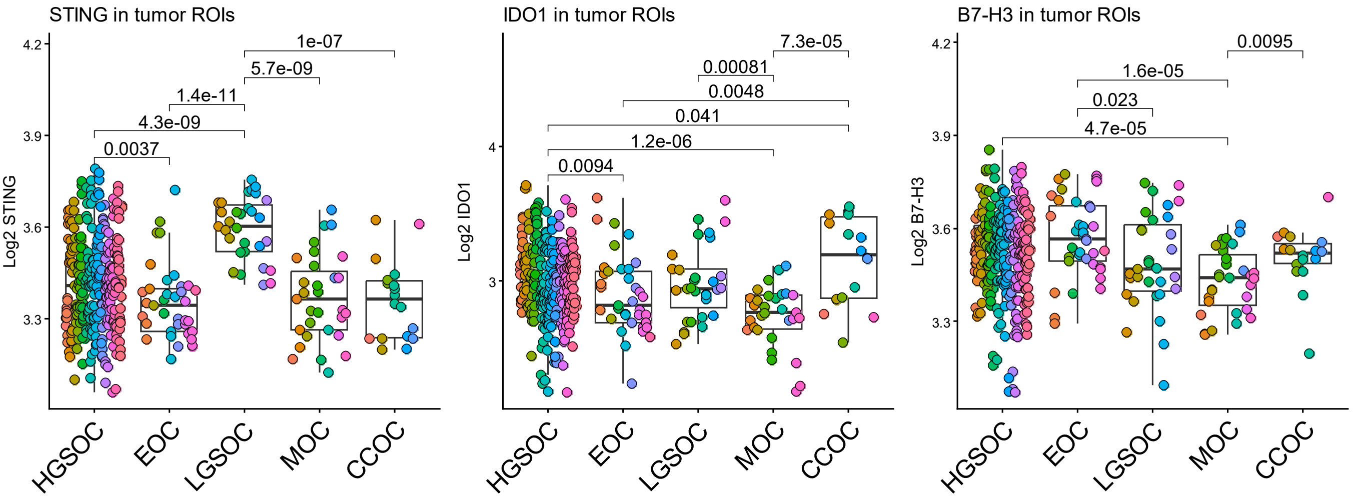

Supplementary Figure 4. Expression of STING, IDO1 and B7-H3 across histotypes. Only tumor ROIs were included. Data is colored by Patient ID. Wilcoxon p-values are shown for significant ($p<0.05$) differences.

Supplementary Figure 5

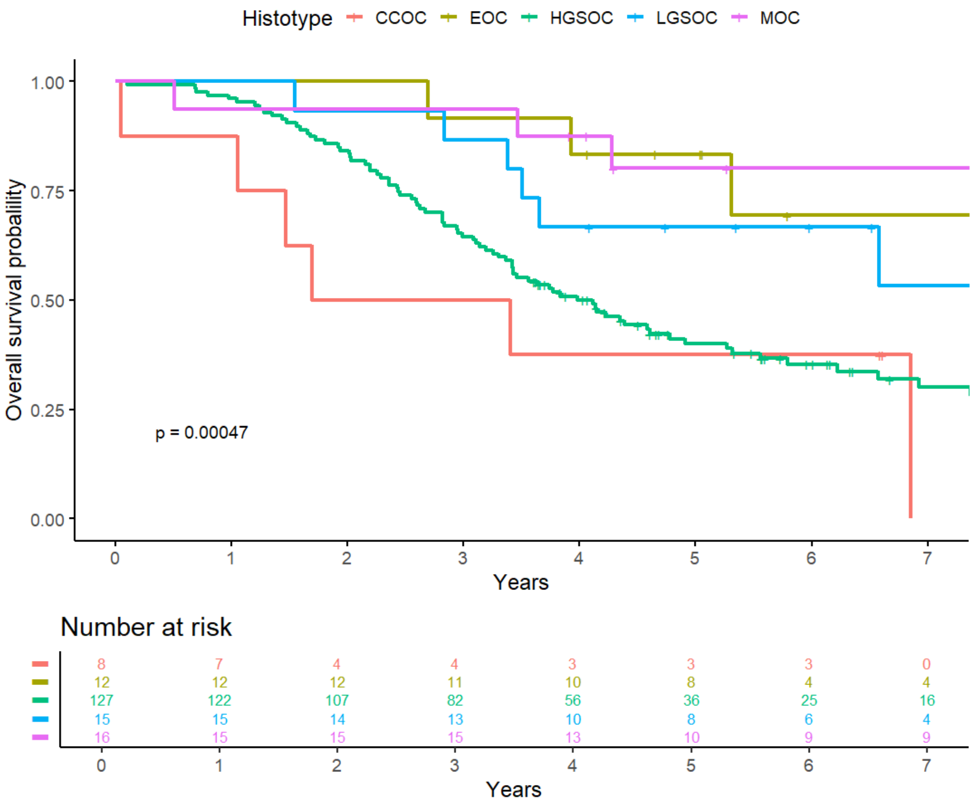

Supplementary Figure 5. Overall survival by OC histotypes. Patients with malignant tumors and for which data on overall survival were available were included.

Supplementary Figure 6

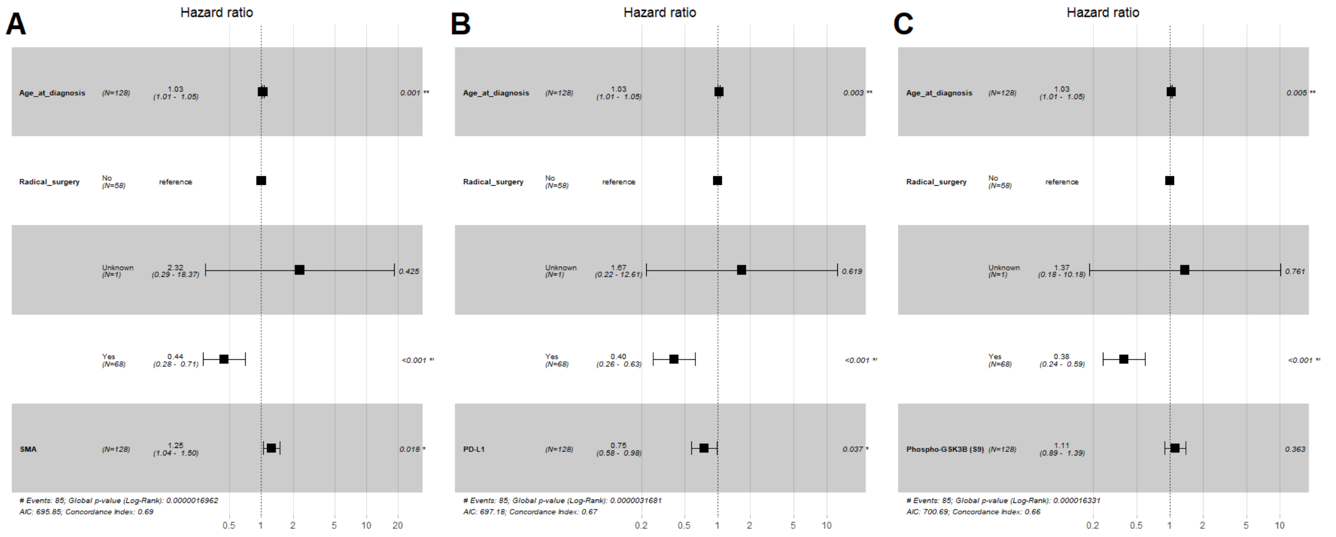

Supplementary Figure 6. Cox proportional hazard models results shown in forest plots. Data is mean expression over patients, HGSOC tumors only and includes variables which are significant in univariate analysis. A) Multivariate analysis with SMA, B) multivariate analysis with PD-L1 and C) multivariate analysis with Phospho-GSK3B (S9).

Supplementary Figure 7

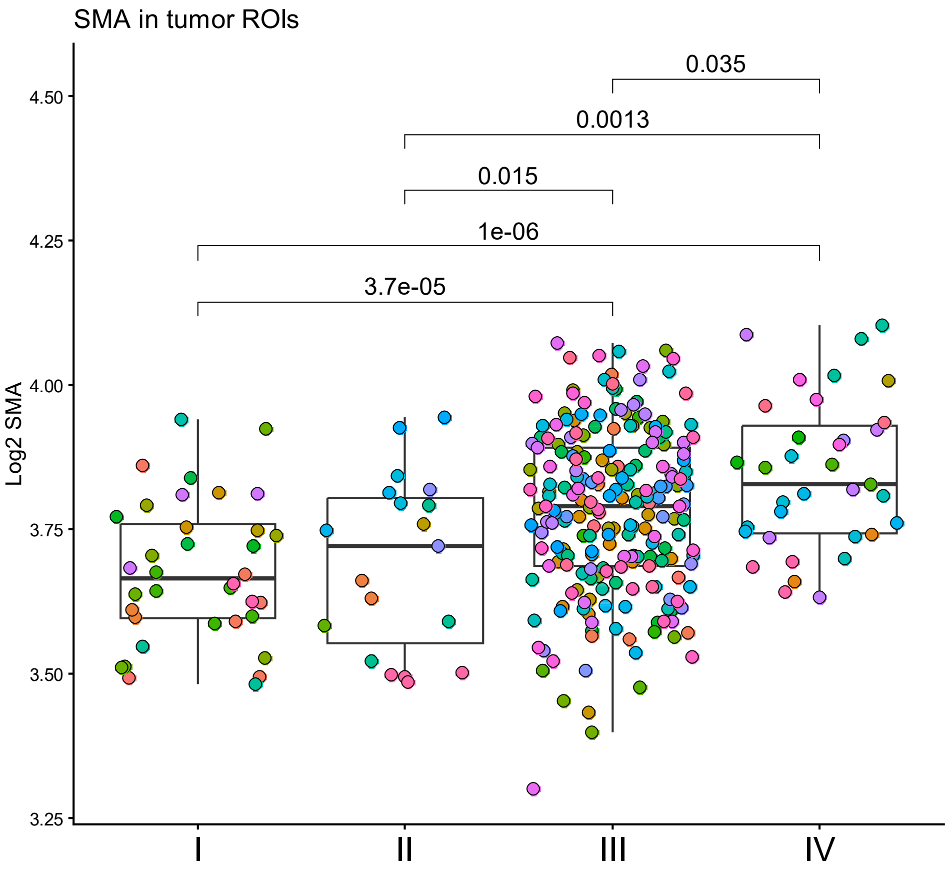

Supplementary Figure 7. Intra-tumoral SMA expression in HGSOC across tumor stages. Only tumor ROIs were included. Data is colored by Patient ID. Significant differences calculated using Wilcoxon test.

Supplementary Figure 8

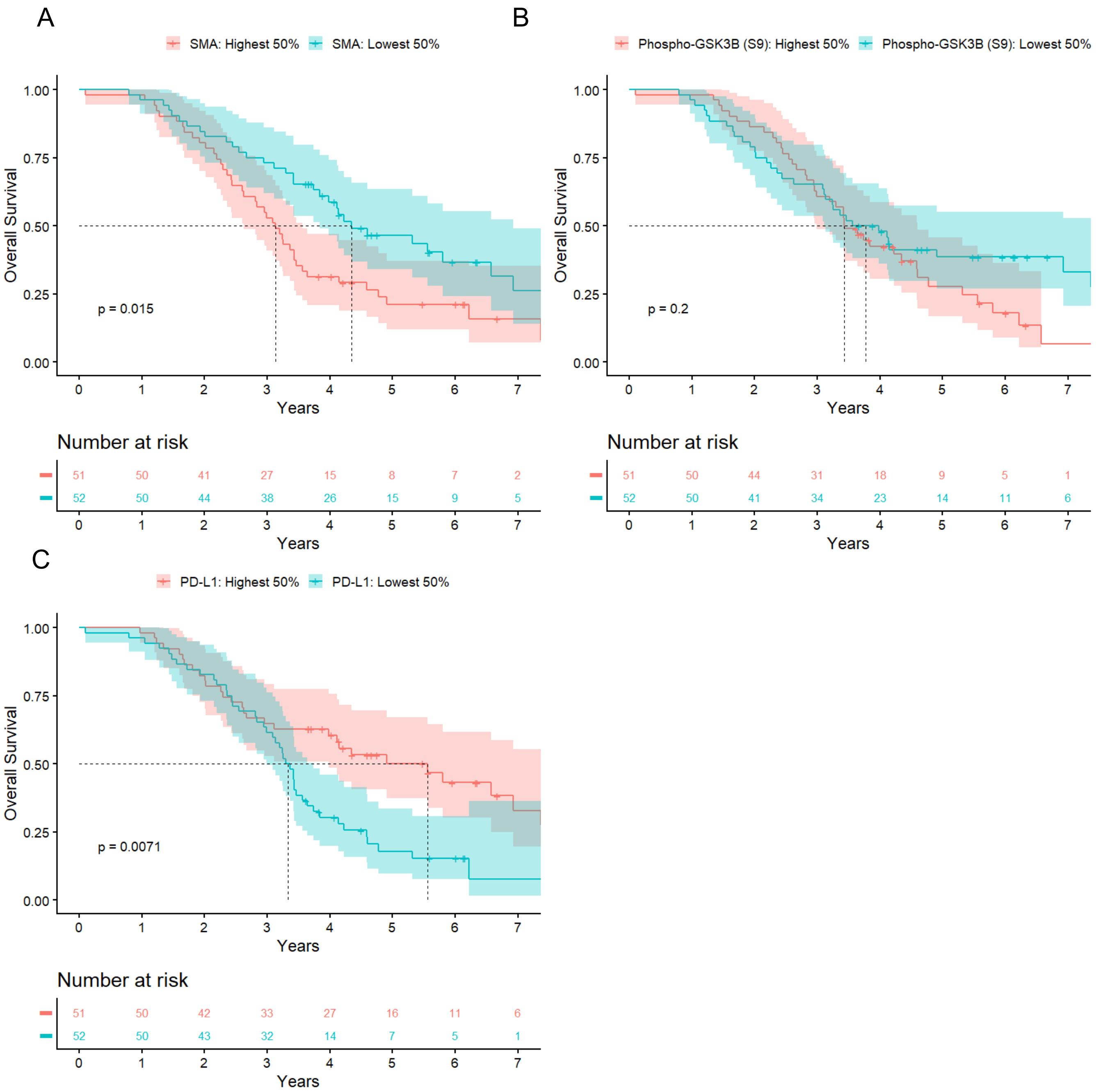

Supplementary Figure 8. Kaplan-Meier analysis of the top three prognostic markers in HGSOC, in late stage (III+IV) HGSOC. Samples were dichotomized by 50th percentile into high/low expression for each respective marker and plotted in relation to overall survival. Only tumor ROIs were included. Dotted line shows median survival. A) SMA; B) Phospho-GSK3B (S9); C) PD-L1

Supplementary Figure 9

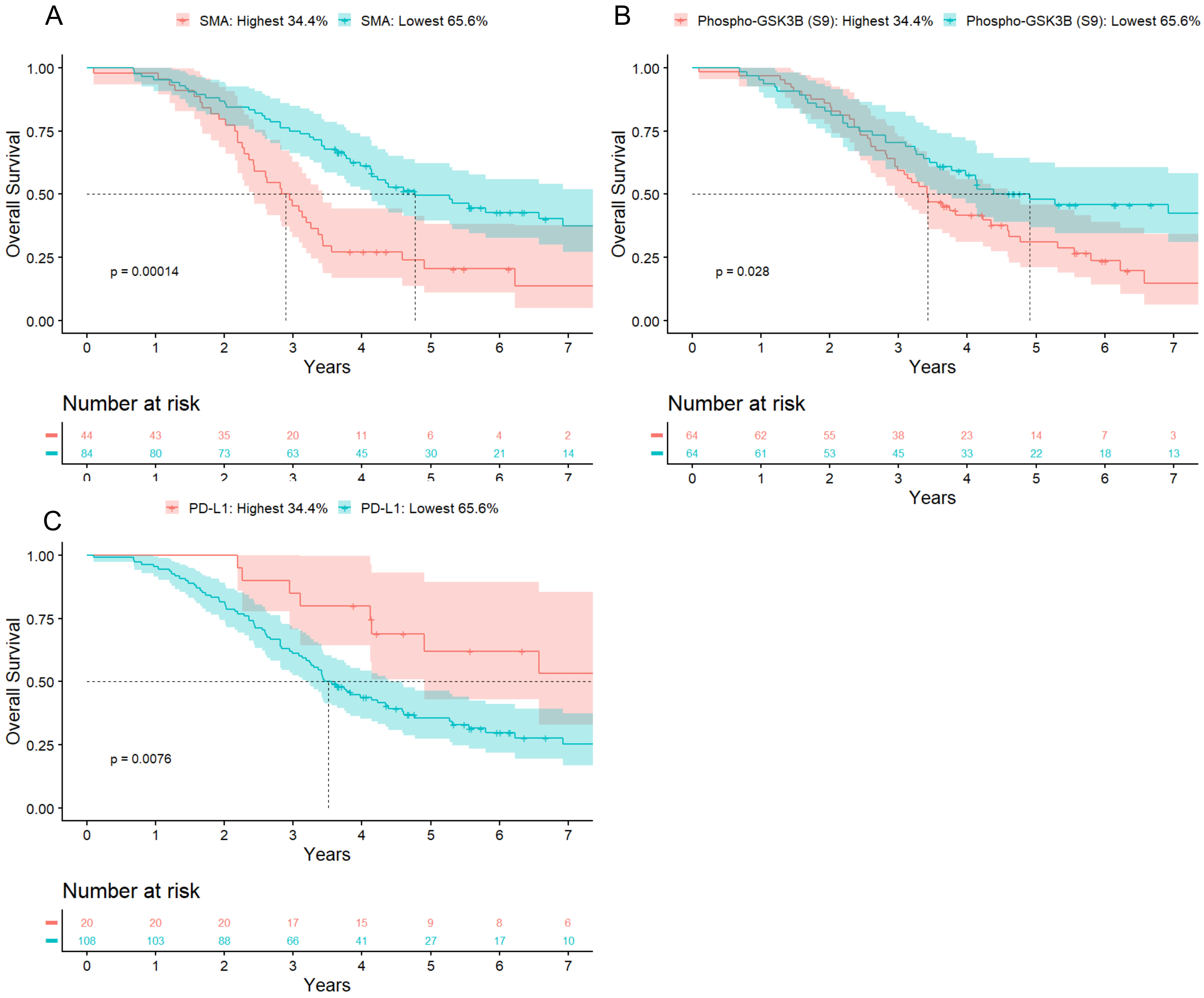

Supplementary Figure 9. Kaplan-Meier analysis of the top three prognostic markers in HGSOC, dichotomized by optimal threshold. Thresholds were defined using log rank tests. Dotted line shows median survival. Only tumor ROIs were included. A) SMA; B) Phospho-GSK3B (S9); C) PD-L1

Supplementary Figure 10

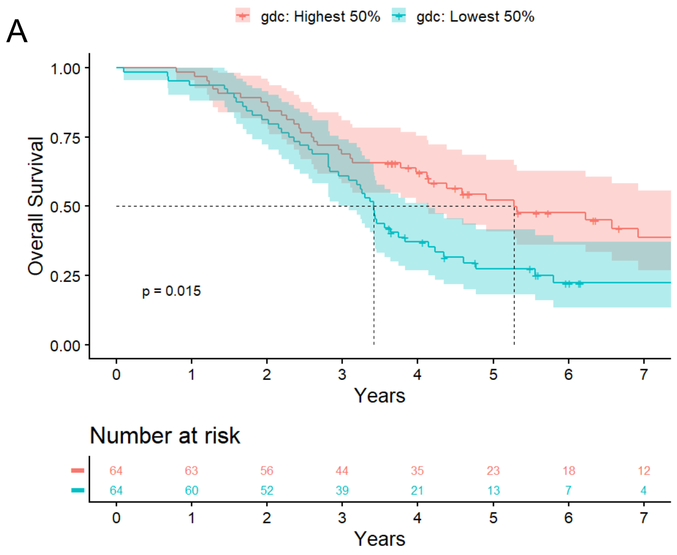

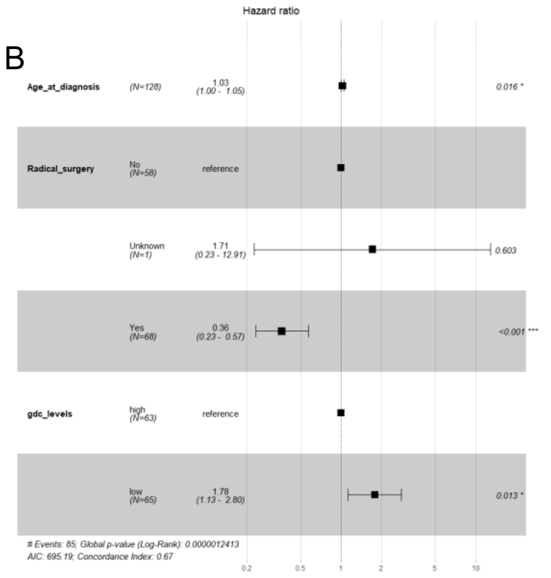

Supplementary Figure 10. Kaplan-Meier analysis of gdc-high/low tumors and a cox proportional hazards multivariate analysis. A) Group degree centrality (gdc) were defined as the ratio of tumor cells in spatial vicinity of at least one CD8+ cells in a set distance (here 30 pixels / 12 µm). Patients were separated into high/low gdc by 50th percentile. Dotted line shows median survival. Only tumor ROIs were included. B) Data is mean expression over patients, HGSOC tumors only of a multivariate analysis with gdc groups and clinically relevant data.

Supplementary Figure 11

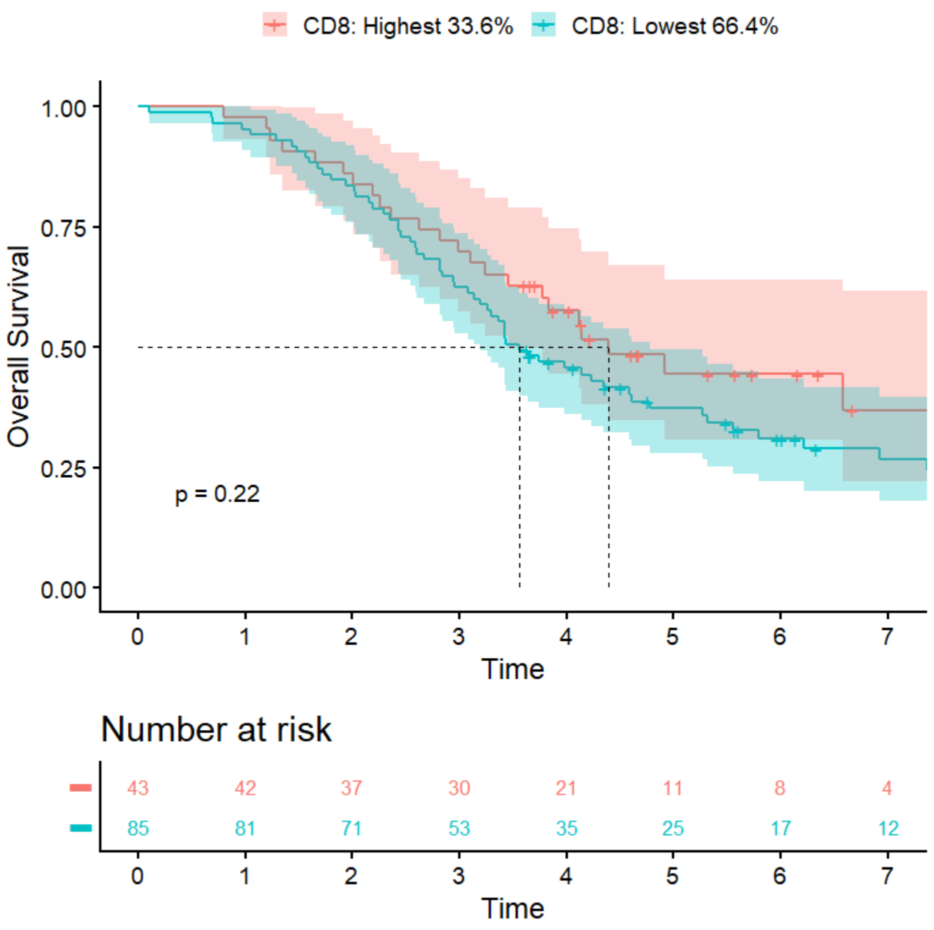

Supplementary Figure 11. Kaplan-Meier analysis of CD8 high/low tumors. Optimal threshold (defined by logrank statistics) was used to divide tumors into high/low CD8. Dotted line shows median survival. Only tumor ROIs were included.
